# Supplementary material for: Single cell analysis in head and neck cancer reveals potential immune evasion mechanisms during early metastasis
Source: Nat Commun. 2023 Mar 27;14:1680. doi: 10.1038/s41467-023-37379-y (PMC10042873; doi:10.1038/s41467-023-37379-y)
Supplement: Supplementary file 1 — Supplementary Information [file 41467_2023_37379_MOESM1_ESM.pdf]

Supplementary Figure 1

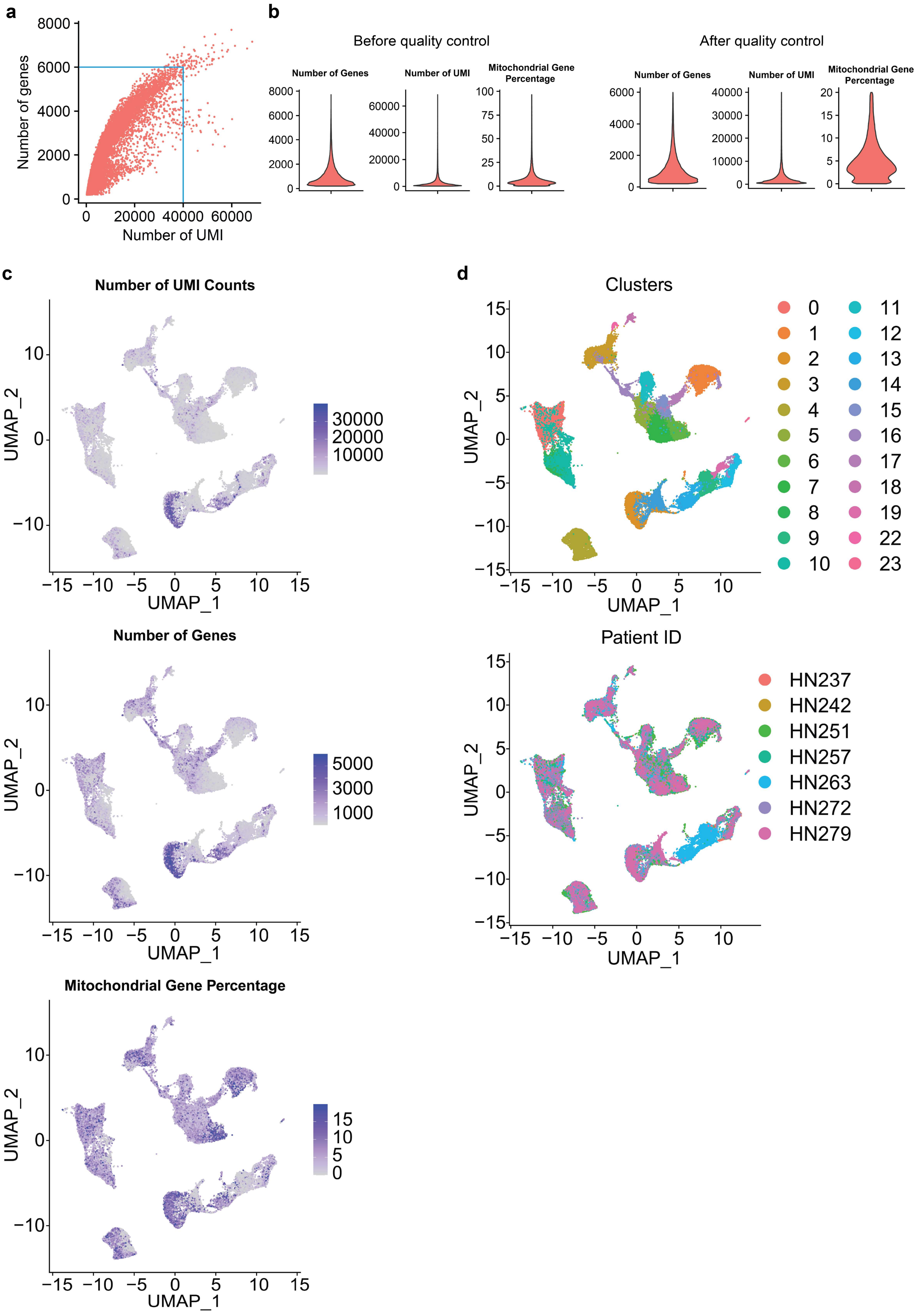

Supplementary Figure 1

e

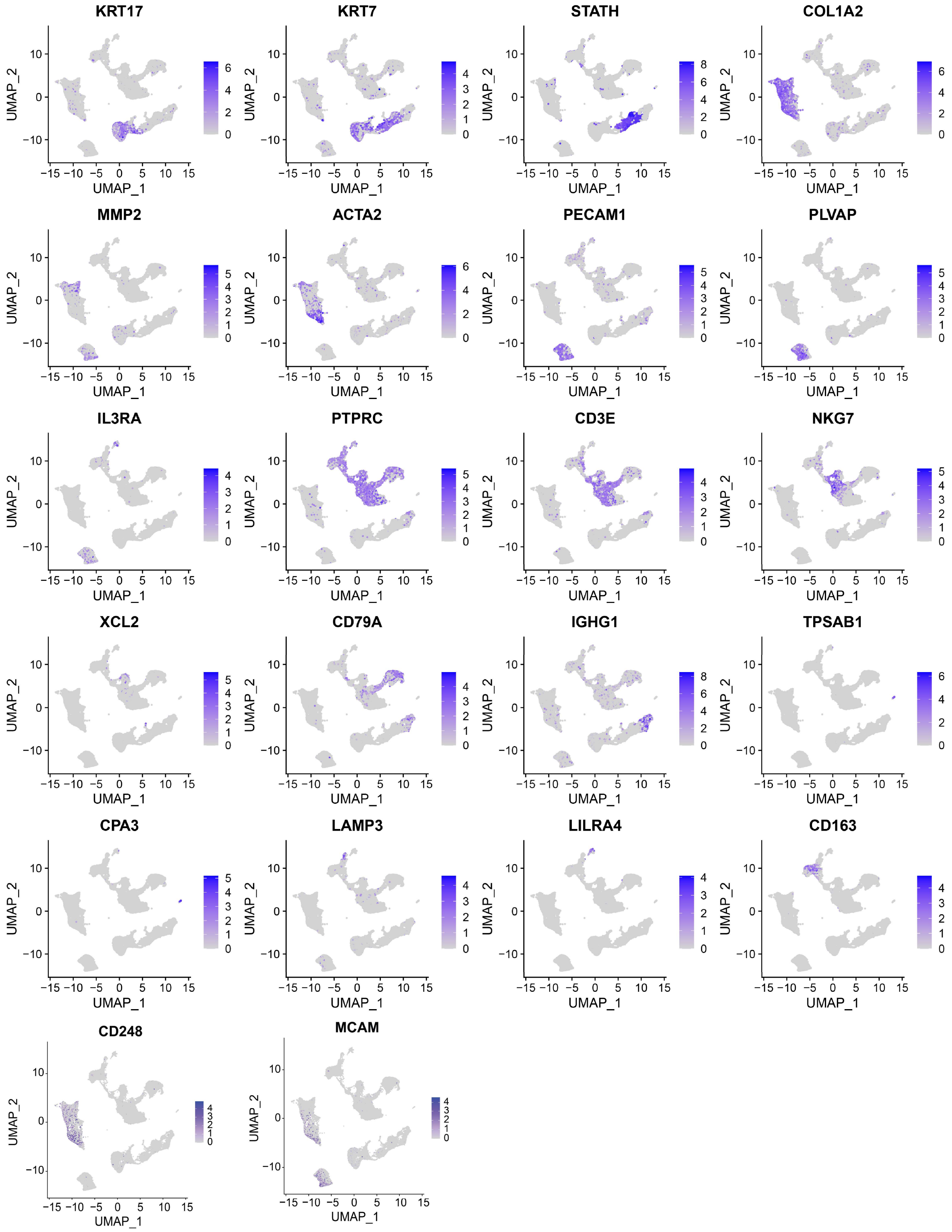

Supplementary Figure 1

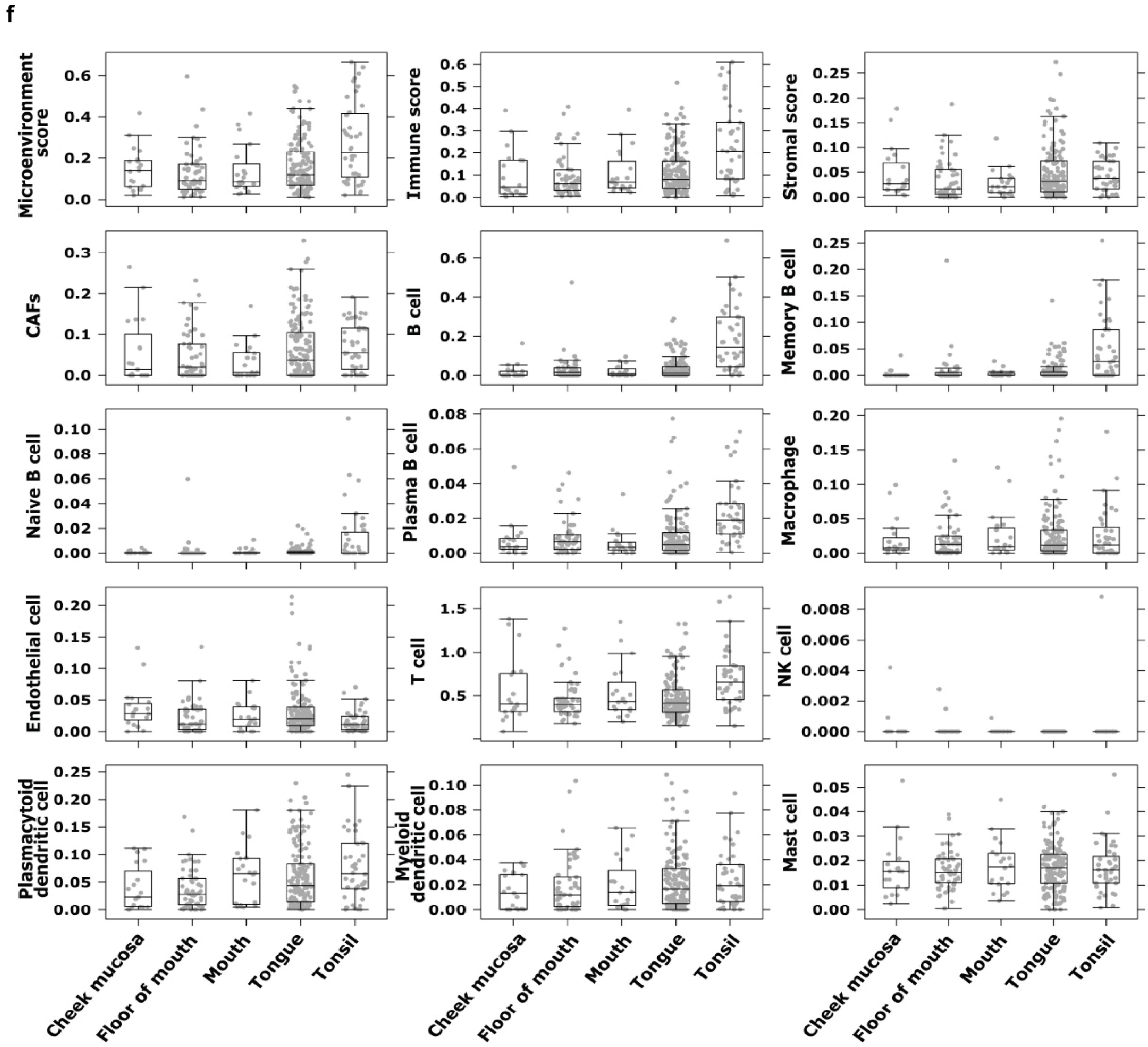

Supplementary Figure 1

g

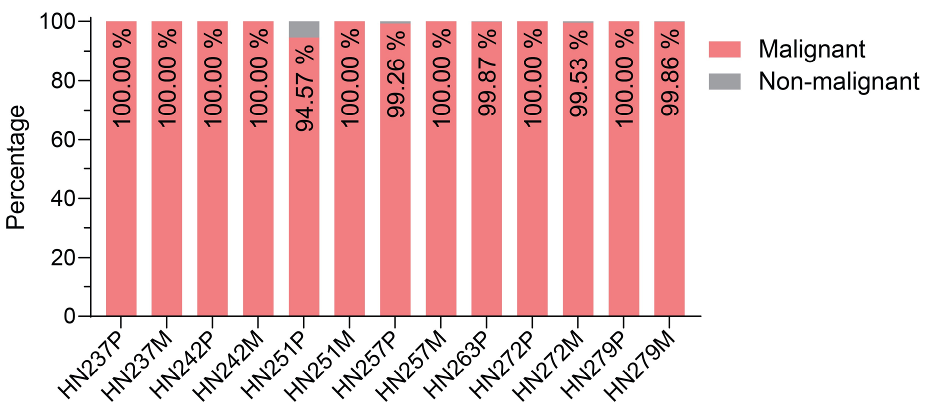

h

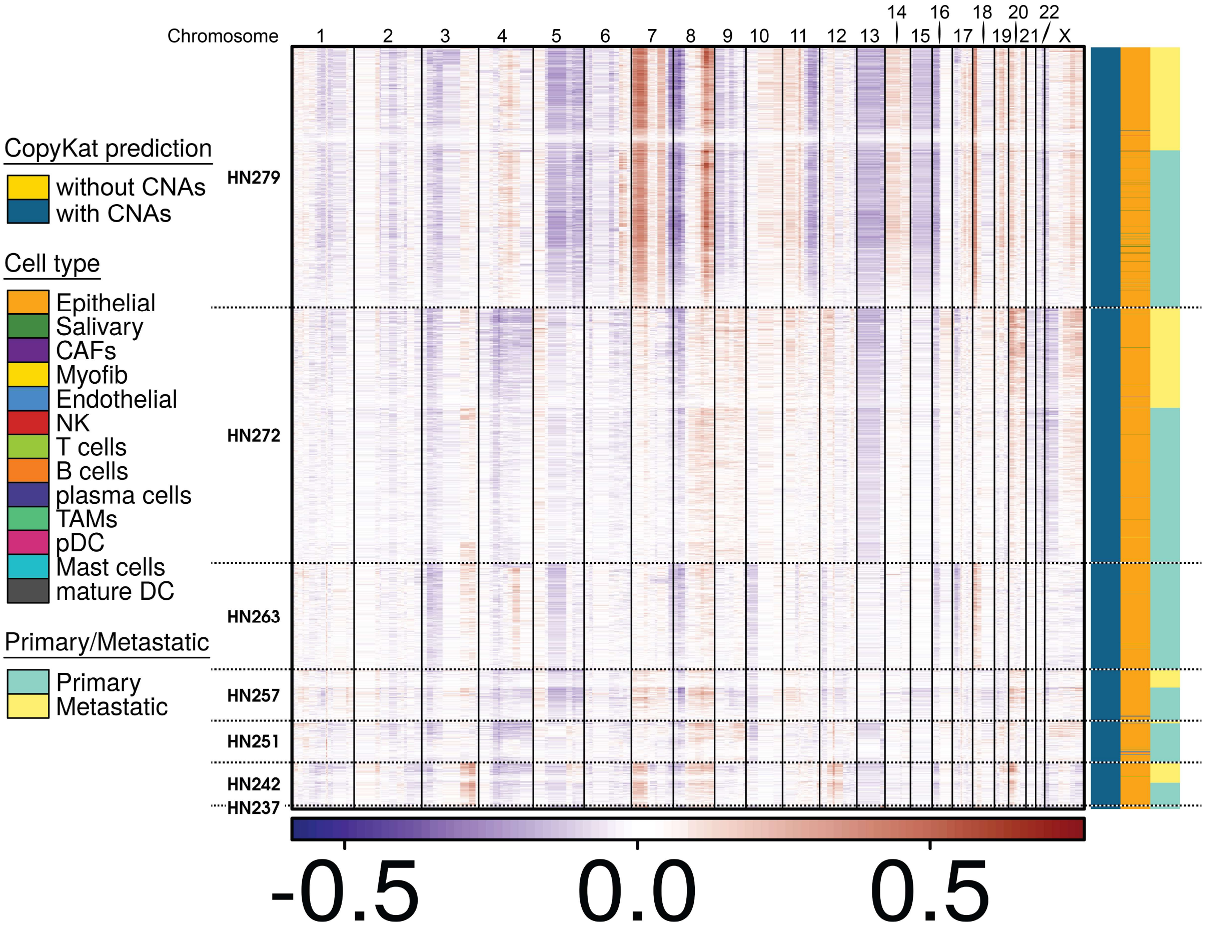

Supplementary Figure 1

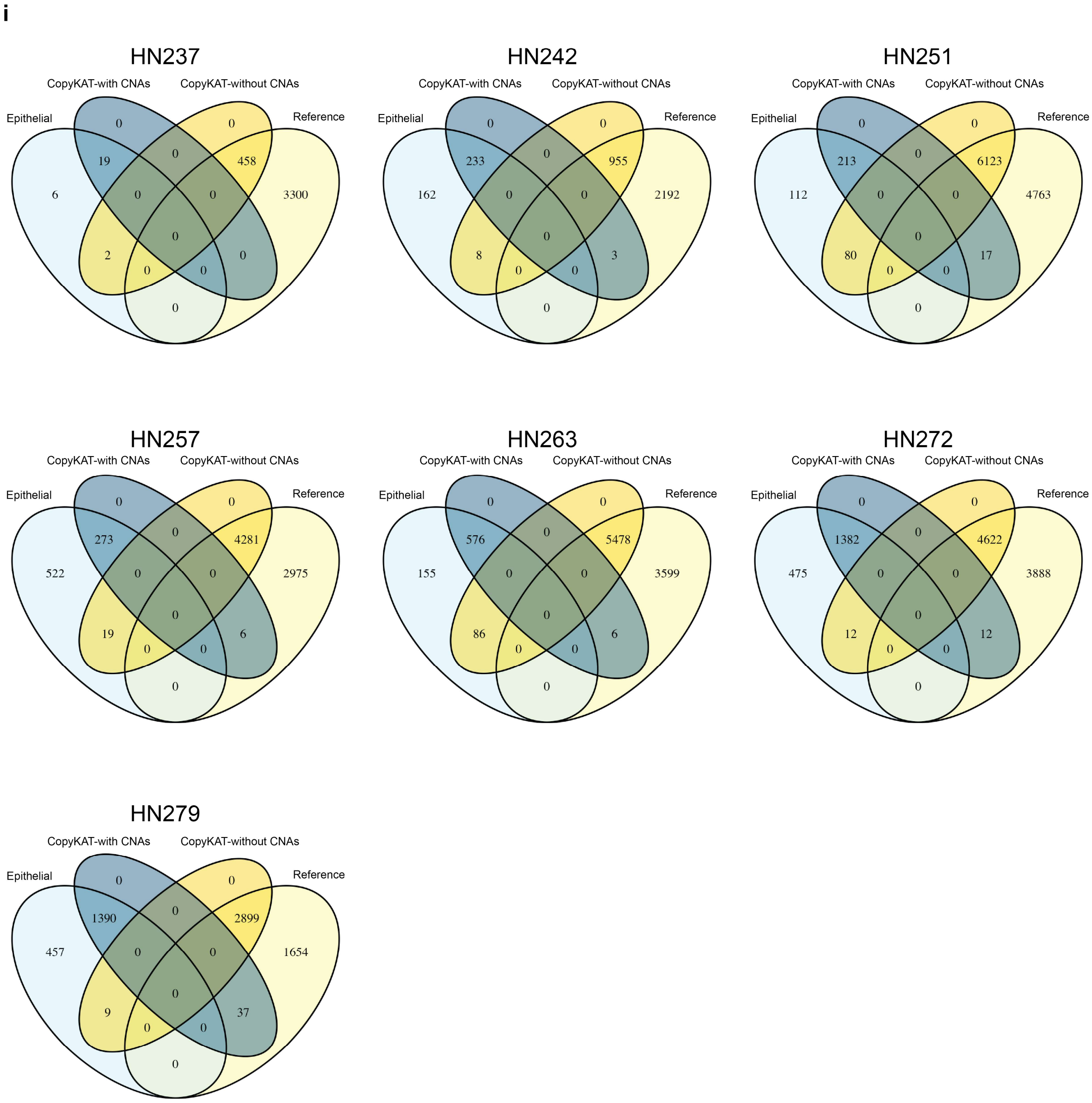

Supplementary Figure 1. a Distribution of each cell by number of genes and UMI as shown by scatterplots. The blue lines indicate the threshold of cutoffs being applied. **b** Violin plots showing the number of genes, number of UMI, and mitochondrial gene percentage in each cell, before (left) and after quality control (right). **c,d** UMAP of all 7 patients derived from HNSCC tumors (n=53,459 cells). Clusters are denoted by **c** (top) number of UMI counts, (mid) number of genes, and (bottom) mitochondrial gene percentage, and **d** (top) Seurat cluster numbers, (bottom) color of the samples. **e** 2D projection of selected genes used for cell type annotation in HNSCC tumors. **f** Comparison of cell composition of tumour sites acquired from TCGA. n=280; Tukey boxplots are shown with the box indicating quartiles with median at middle, and the whiskers drawn at the lowest and highest points within 1.5 interquartile range of the lower and upper quartiles, respectively. **g** Percentage of malignant and non-malignant epithelial cells of each sample as predicted by InferCNV. N=6,191 cells. **h** Chromosomal gains and losses prediction for malignant epithelial cells by CopyKAT using non-malignant cells from respective samples as controls. Cyan indicates primary malignant epithelial; yellow indicates metastatic lymph node malignant epithelial; sample identities on the left y-axis, chromosome numbers on the top x-axis. CNAs indicates copy number alterations. **i** Venn diagrams

Supplementary Figure 1

comparing the overlapping epithelial (malignant cells called by InferCNV), reference (non-malignant cells by InferCNV) and CopyKAT predicted cells with or without CNAs.

Supplementary Figure 2

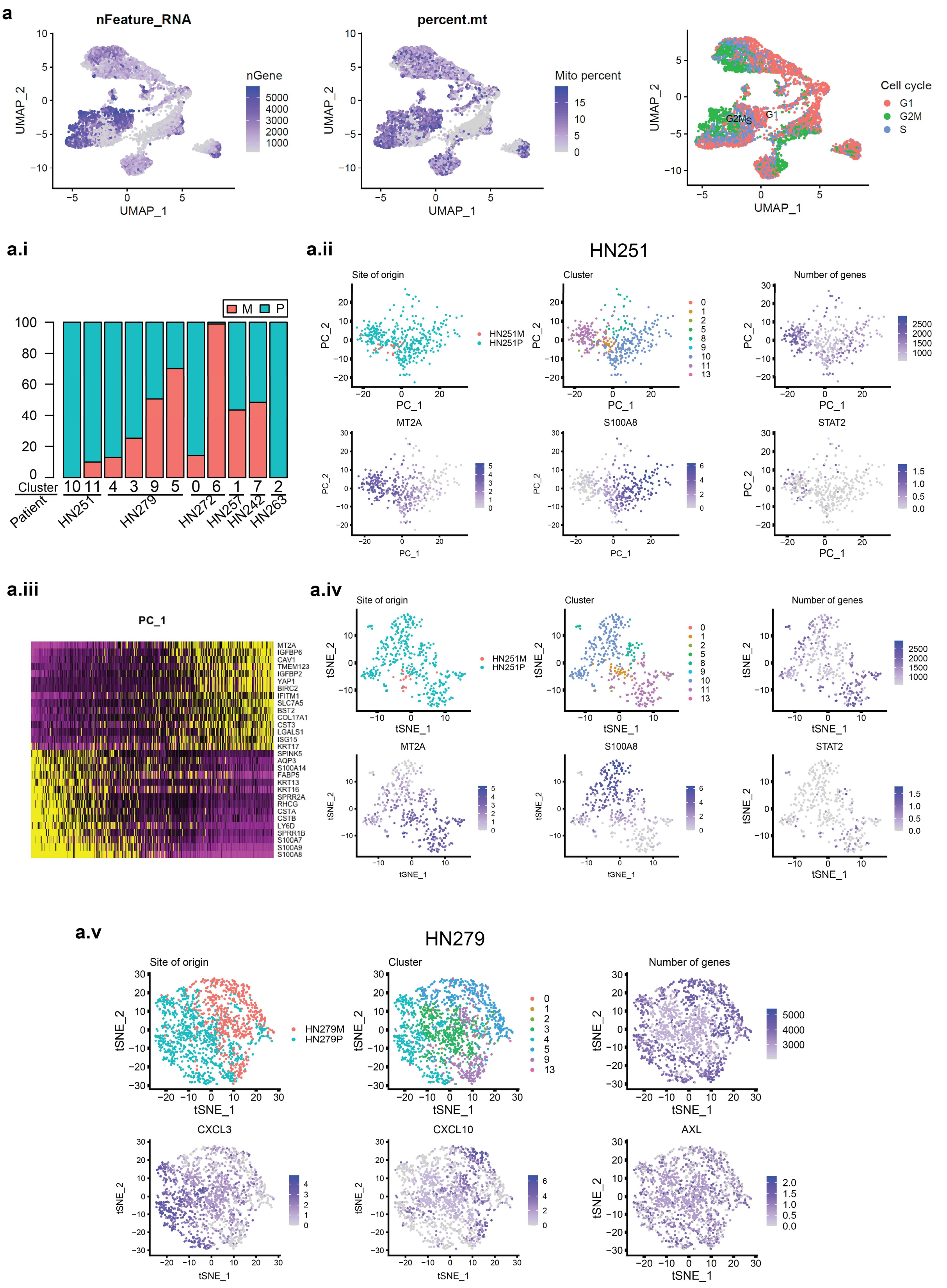

Supplementary Figure 2

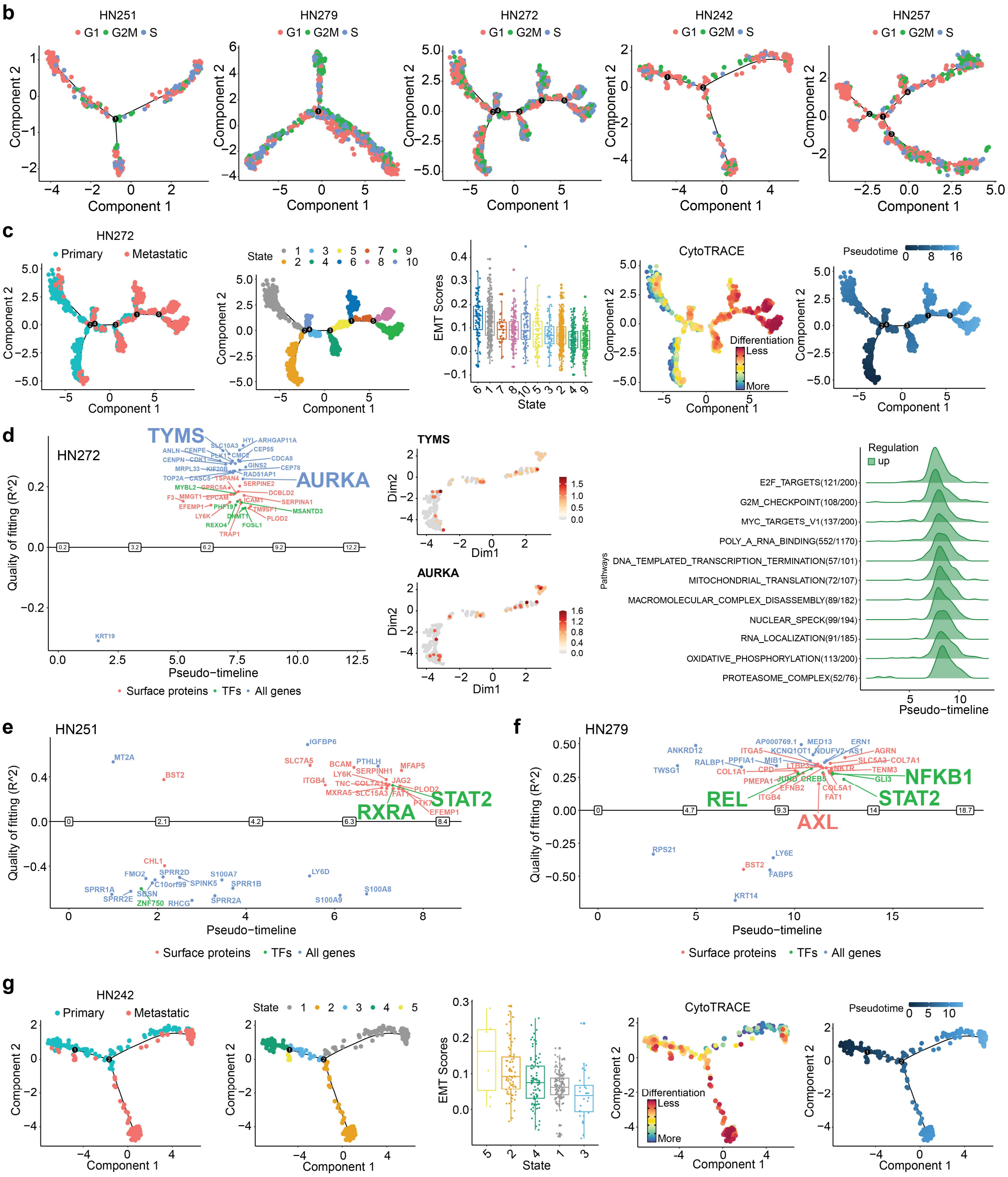

Supplementary Figure 2

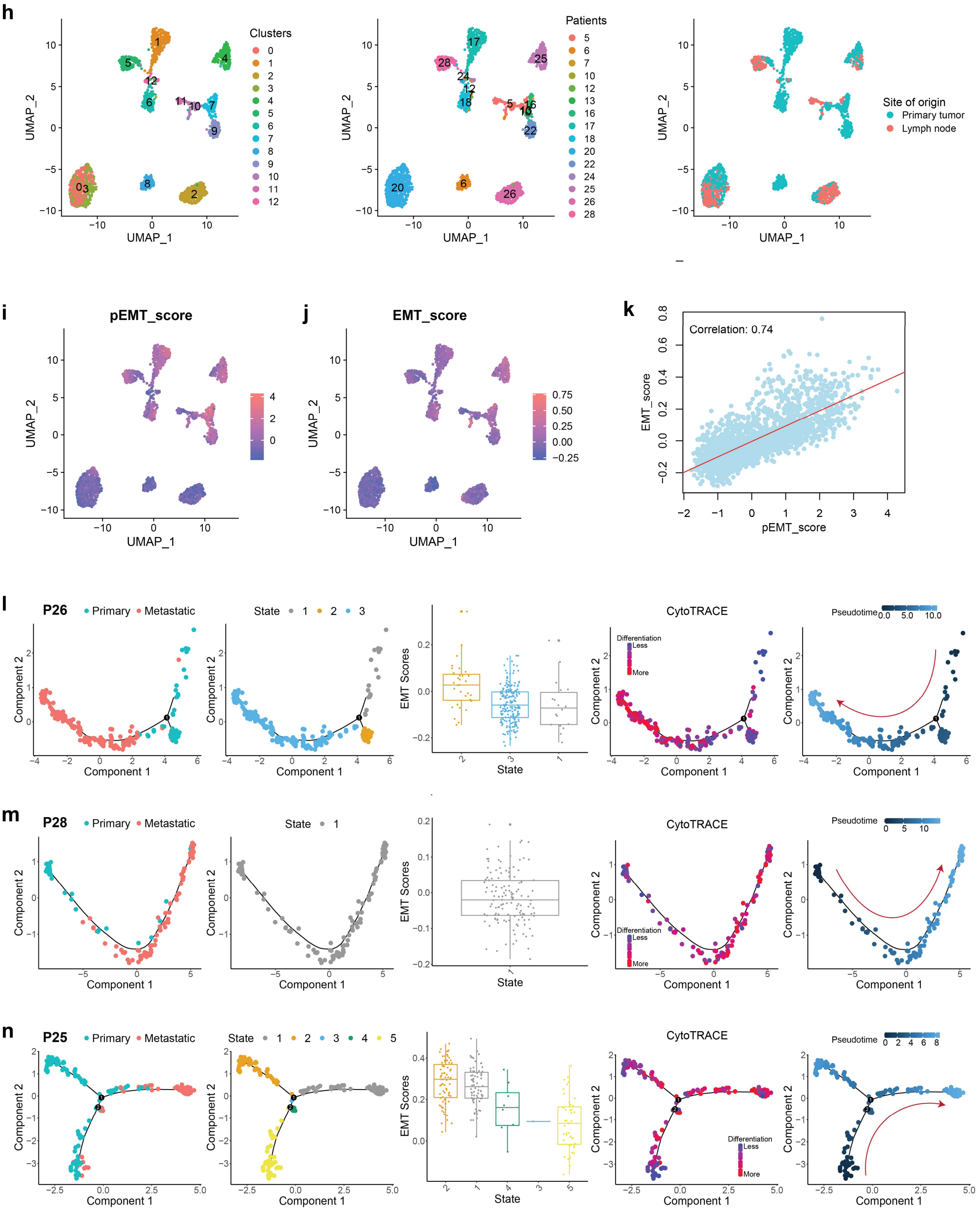

Supplementary Figure 2

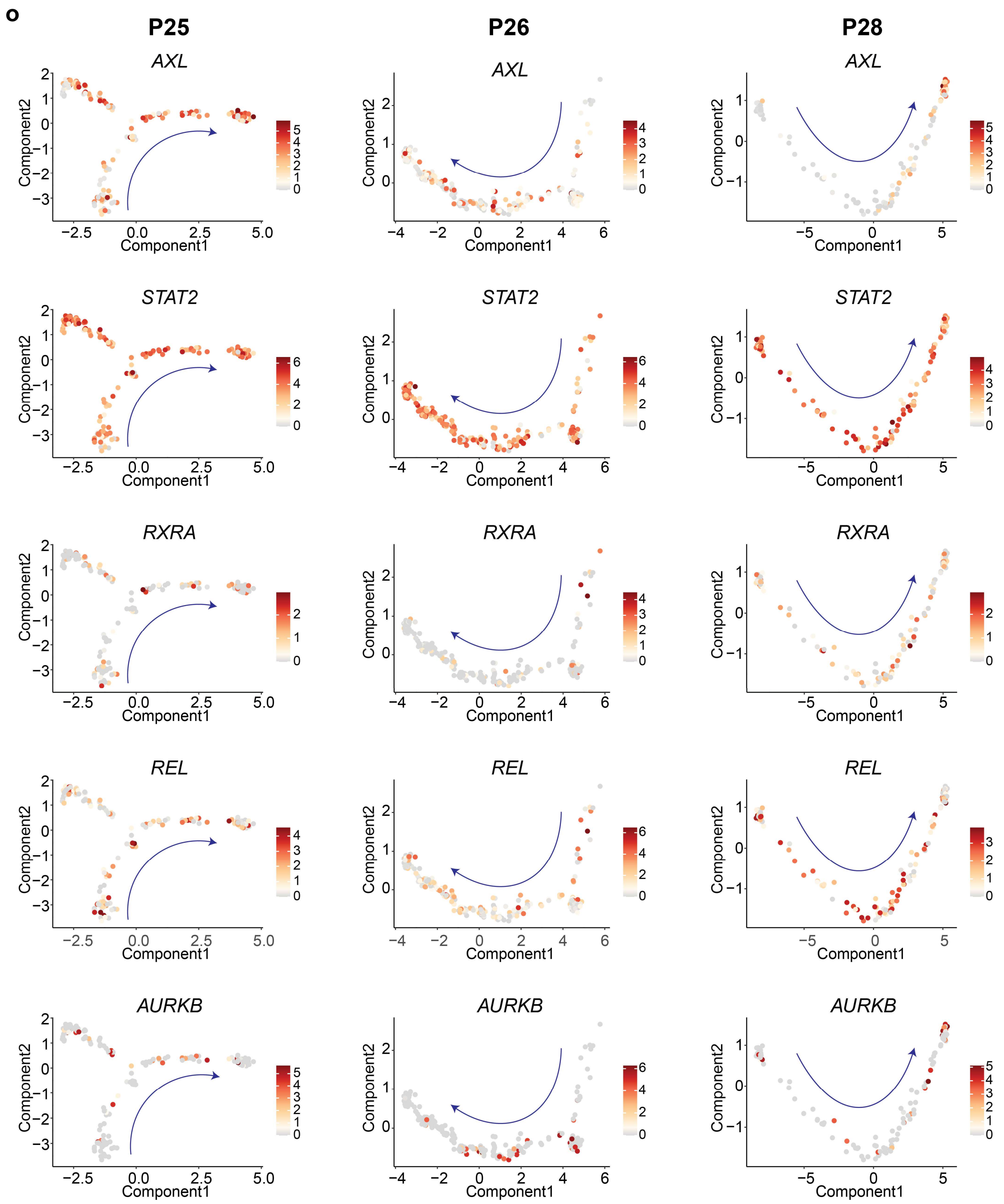

Supplementary Figure 2

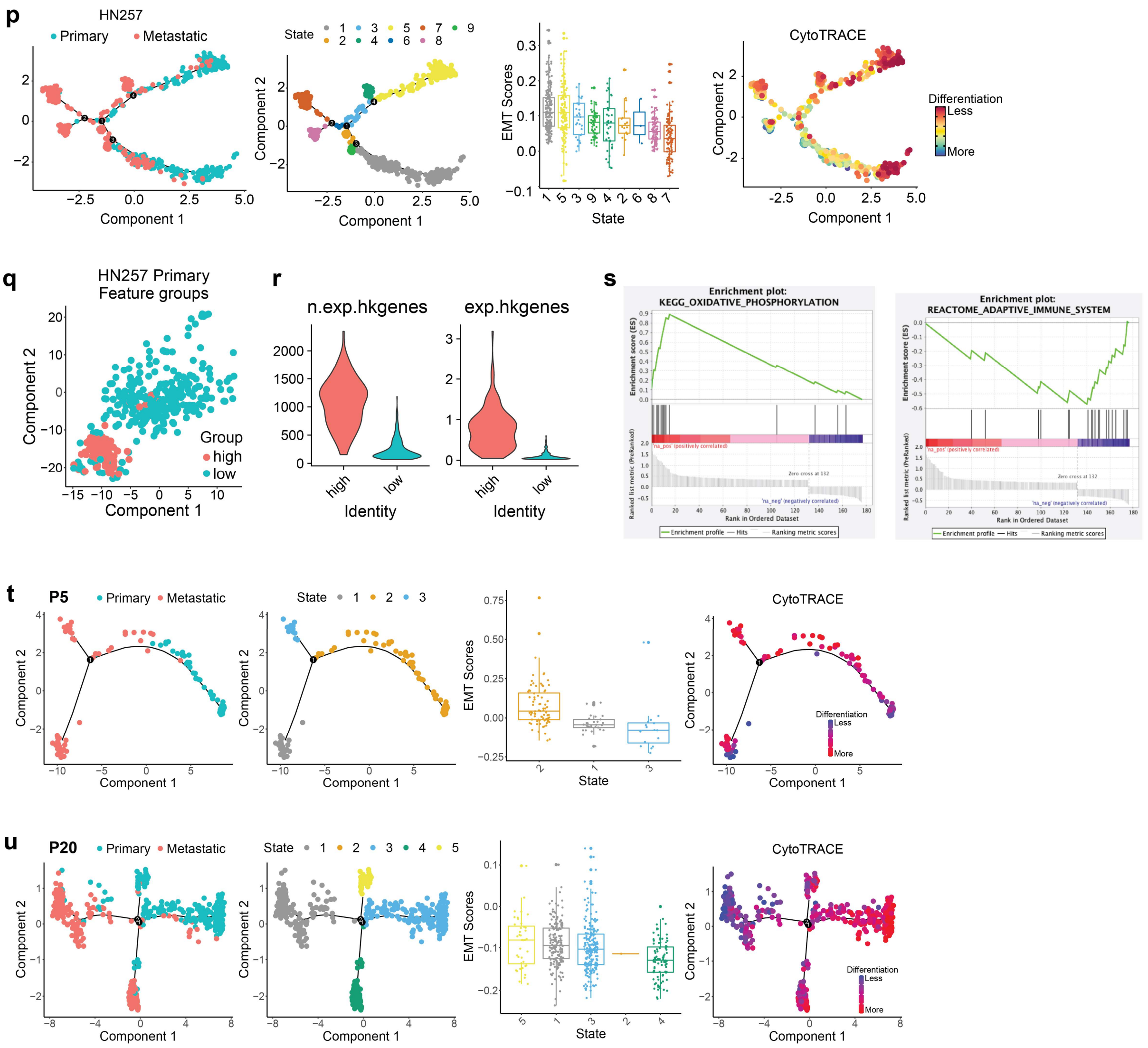

Supplementary Figure 2. a UMAP of all 6,115 malignant epithelial cells of HNSCC tumors. Clusters are denoted by **a** (left) number of genes, (mid) percentage of mitochondrial genes and (right) cell cycle phase detected in each cell by Seurat. **a.i** Barplot showing the percentage of cells from primary (P) or metastatic (M) site of each cluster. The patient ID is the major composition for the corresponding clusters. Cluster numbers on the x-axis correspond to those of Figure 2a. **a.ii** PCA plots of cells from patient HN251 (n=405) colored by site of origin, cluster and number of genes (upper panel). Gene expression plots in PCA embedding for gene MT2A, S100A8 and STAT2 as denoted by the title of each plot. **a.iii** Heatmap of top differentially expressed genes along PC1 of **a.ii**. **a.iv** t-SNE plots of cells from patient HN251 (n=405) colored by site of origin, cluster and number of genes (upper panel). Gene expression plots in t-SNE embedding for gene MT2A, S100A8 and STAT2 as denoted by the title of each plot. **a.v** t-SNE plots of cells from patient HN279 (n=1,856) colored by site of origin, cluster and number of genes (upper panel). Gene expression plots in tSNE embedding for gene CXCL3, CXCL10 and AXL as denoted by the title of each plot. **b** Monocle trajectory projection colored by cell cycle phases derived using Seurat function for each patient. **c** Trajectory construction for the derivation of pre-metastatic populations in HN272 malignant epithelial cells using Monocle2 (n=1,611 cells), colored by (from left to right) origin of tumor sites, monocle branch states, boxplot of EMT score for each branch state, CytoTRACE score, monocle pseudo-time and the root was labelled as state 2. **d**

Supplementary Figure 2

Visualization of GeneSwitches results of top 70 switching genes for patient HN272 primary epithelial cells on trajectory along monocle state 2-4-6-7 (left). Expression plots of *TYMS* and *AURKA* of HN272 primary epithelial cells on this trajectory (middle). Density plots of switching genes for significantly over-represented functional ontologies determined by GeneSwitches for patient HN272 primary epithelial cells (right). N= 472 cells. **e,f** Visualization of GeneSwitches results of top 70 switching genes for patient HN251 (based on the primary epithelial cells on trajectory from monocle state 3 to 1) and HN279 (based on the primary epithelial cells on trajectory from monocle state 1 to 3). **e,f** For GeneSwitches, x-axis is the corresponding monocle pseudo-time and y-axis is the confidence level. Genes are colored by various sets of known proteins, including surface proteins and transcription factors. **g** Trajectory construction for the derivation of pre-nodal populations in HN242 malignant epithelial cells using Monocle2 (n=343 cells). **h** UMAP of all 2,076 malignant epithelial cells collected from HNSCC tumors derived from Puram *et al*[1]. Clusters are denoted by (left) Seurat cluster numbers, (middle) patient numbers, (right) site of tissue origins. **i,j** UMAP of malignant cells showing **i** pEMT score of genes associated with partial EMT in each cell as described in Puram *et al.* and **j** EMT score of genes associated with EMT in each cell as described in this paper. **k** Scatter plots with a liner regression line showing the relationship between pEMT score as described in Puram *et al* and EMT score as described in this paper. Pearson correlation between these two scores was as high as 0.74. **l-n** Trajectory construction using Monocle2 for the derivation of pre-nodal malignant populations in **l** p26 (n=267 cells), **m** p28 (n=138 cells) and **n** p25 (n=209 cells). **o** Gene expression plots of potentially actionable genes related to EMT identified in this paper for patient p26, p28 and p25. **l-o** Single cell RNA-seq data of malignant cells derived from Puram *et al*[1]. **p** Trajectory construction for the derivation of pre-nodal populations in HN257 malignant epithelial cells using Monocle2 (n=706 cells). **q** t-SNE plot of HN257 primary tumor cells colored by high (red) or low (blue) cytotrace scores in each tumor cells (see Figure 2g), **r** as indicated by violin plots showing high expression number of (n.exp.hkgenes) and high expression level (exp.hkgenes) of housekeeping genes. **s** GSEA plots of top up- and down-regulated pathways. **t,u** Trajectory construction using Monocle2 for the derivation of pre-nodal malignant populations in **s** p5 (n=132 cells) and **t** p20 (n=570 cells). **t,u** Single cell RNA-seq data of malignant cells derived from Puram *et al*[1]. Box plots in **c, g, l-n, p, t, u** centered at the median with hinges at 1st and 3rd quartiles and whiskers drawn from hinges to the lowest and highest points within 1.5 interquartile range.

Supplementary Figure 3

a

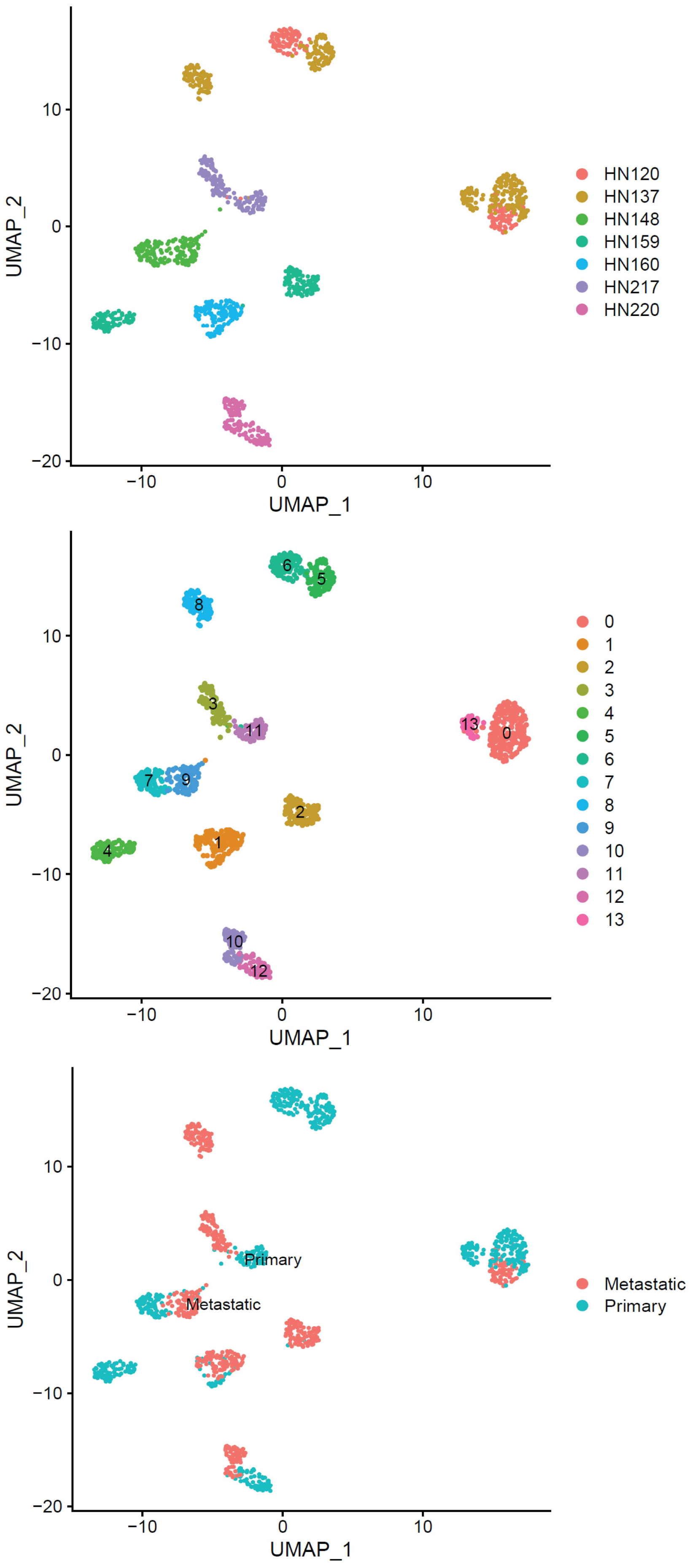

Supplementary Figure 3

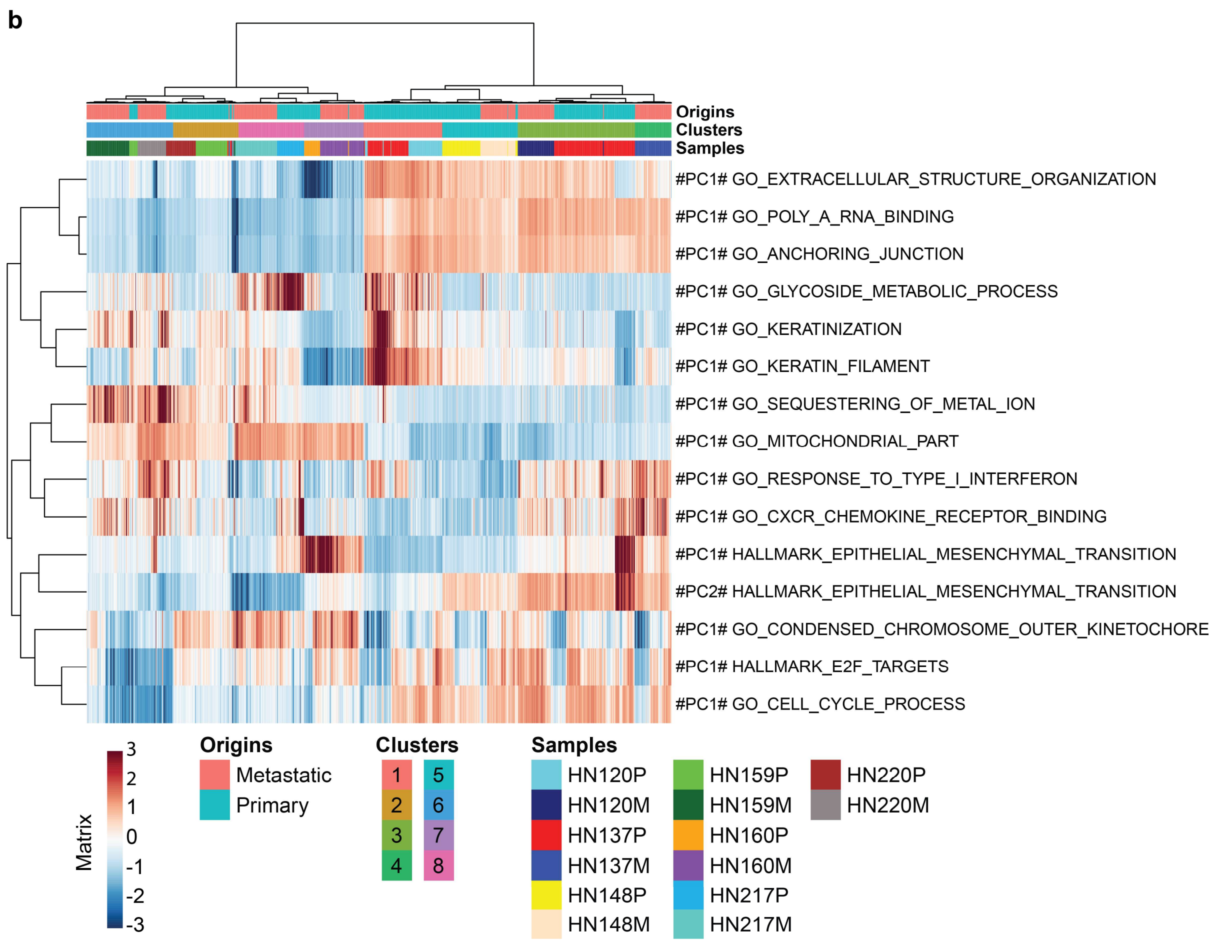

Supplementary Figure 3

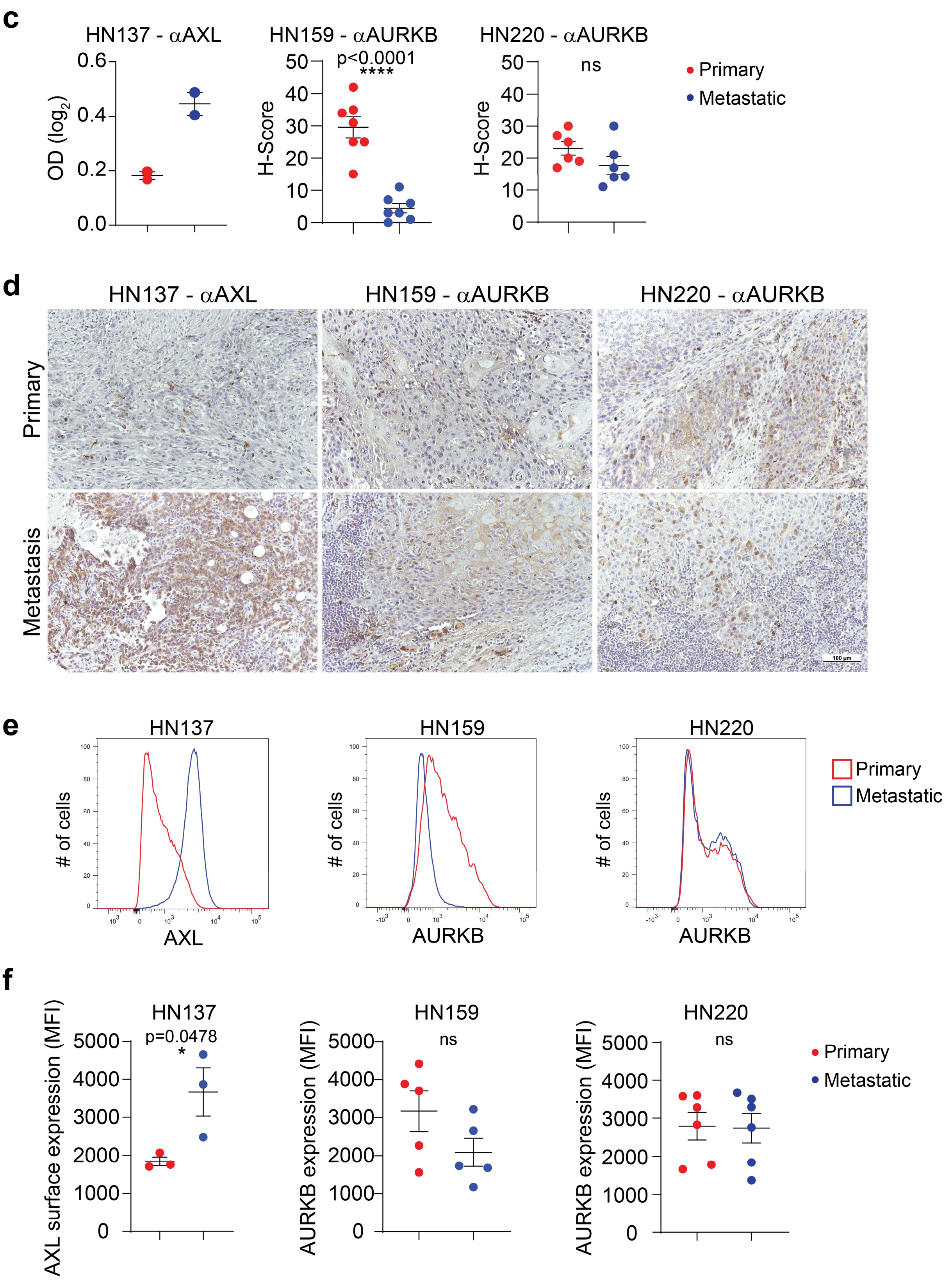

Supplementary Figure 3

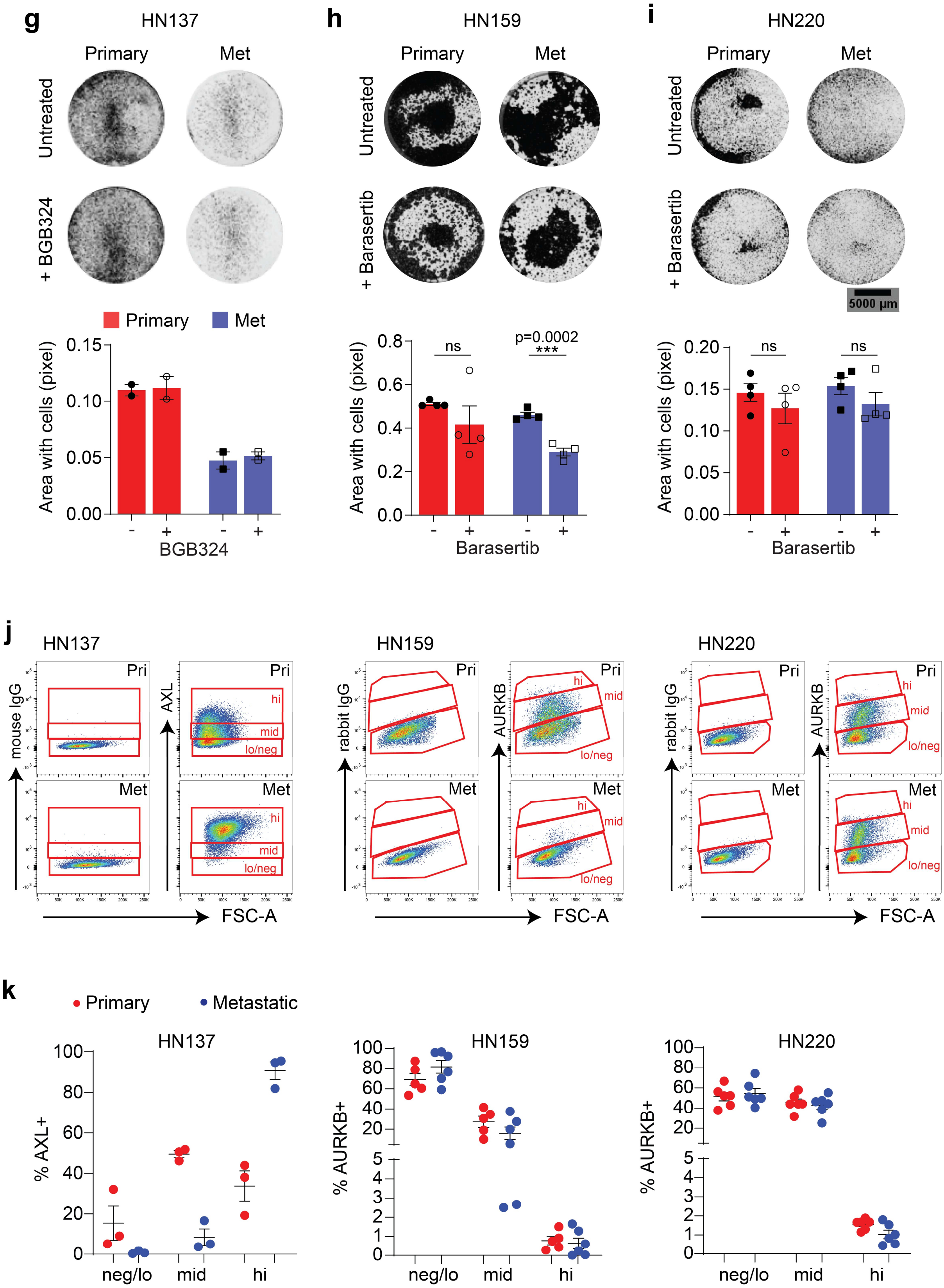

l

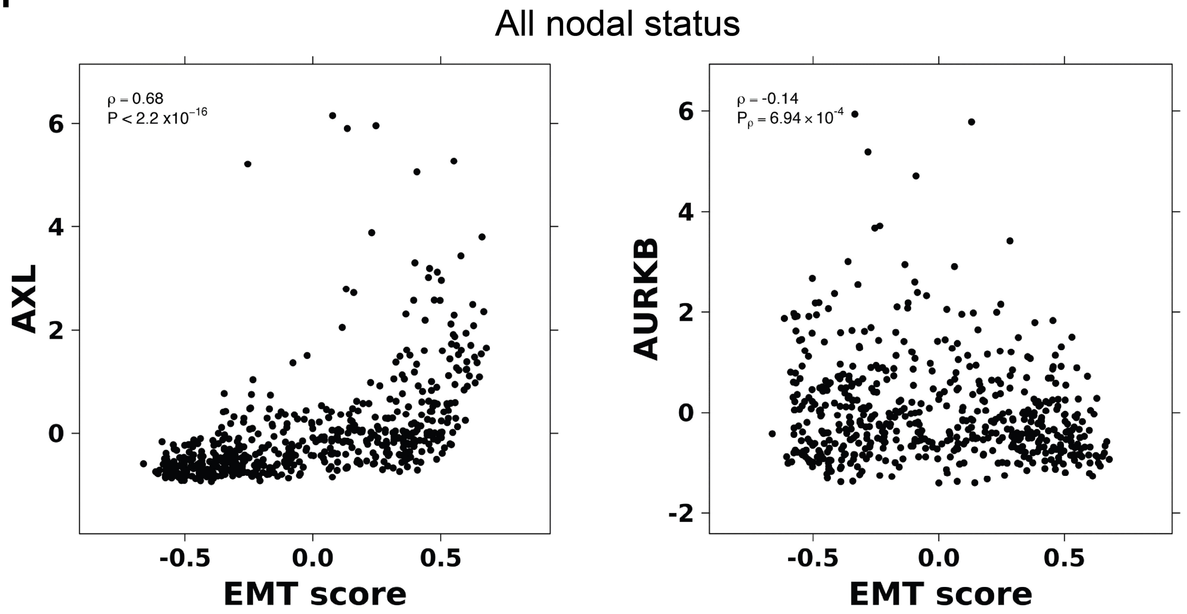

m

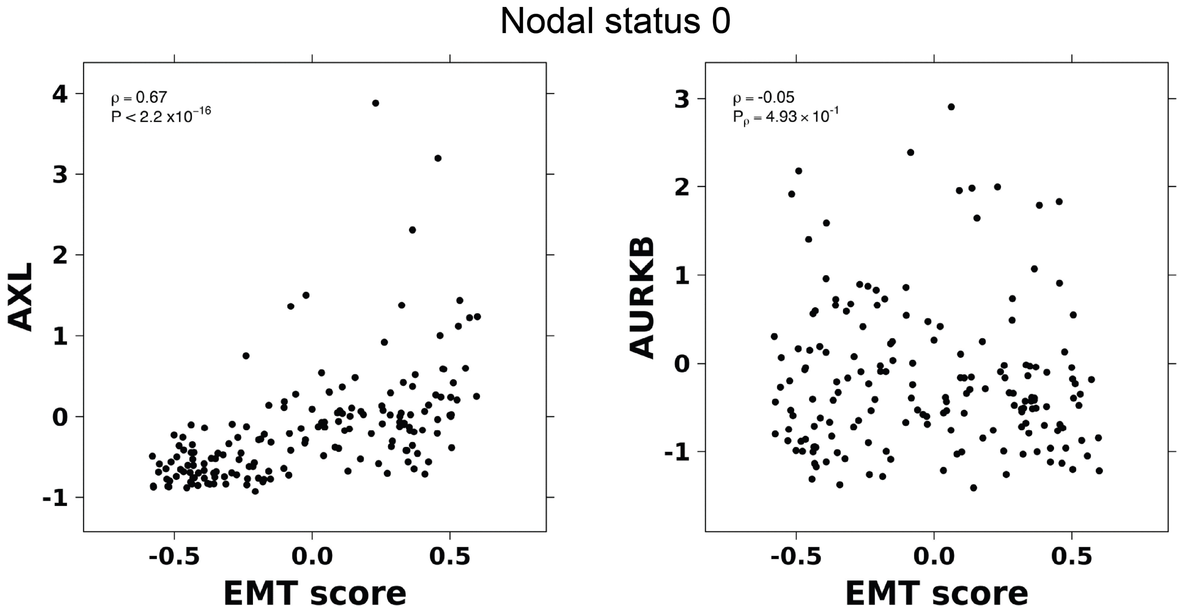

Supplementary Figure 3. a UMAP of all patients derived cell cultures from HNSCC tumors. Clusters are denoted by (top) colour of the samples, (middle) Seurat cluster numbers, (bottom) site of tissue origins. N=1,317 cells. **b** Heatmap of differentially expressed genes (rows) between cells organized by top 15 Hallmark and Gene Ontology (GO) gene sets (columns). Bars on the top of the heat map indicate the site of sample origins, clusters and patient samples. **c** Quantification of primary and metastatic culture cells stained positive for AXL (HN137) and AURKB (HN159 and HN220) by immunocytochemistry as seen in Figure 3D. n=2 pairs for HN137 performed in 2 independent experiments, n=7 pairs for HN159 performed in 7 independent experiments and n=6 pairs for HN220 performed in 6 independent experiments. Black lines and error bars represent mean ± SEM. Red dot represents primary and blue dot represents metastatic. ****p<0.0001 indicate significant difference by unpaired two-tailed t test when compared to control, not corrected for multiple comparisons, ns

not significant. Statistical analysis was not performed for HN137 as the experiment was repeated only twice. **d** Immunohistochemistry micrographs representing staining of AXL (brown) in HN137 (n=6), and AURKB (brown) in HN159 (n=7) and HN220 (n=8) of patient tumor tissues, counter-stained with haematoxylin (blue). Scale bar indicates 100 µm. **e** Representative histograms show the expression of surface AXL on HN137, and intracellular AURKB of HN159 and HN220 as determined by flow cytometry. Primary (red) and metastatic (blue). **f** MFI expression of surface AXL on HN137 and intracellular AURKB of HN159 and HN220 by flow cytometry. n=3 pairs for HN137 performed in 3 independent experiments, n=5 pairs for HN159 performed in 5 independent experiments, and n=6 pairs for HN220 performed in 6 independent experiments. Red dot represents primary and blue dot represents metastatic. Black lines and error bars represent mean ± SEM. *$p \leq 0.05$ indicates statistical significance by unpaired two-tailed t test, ns not significant. **g-i** (Top) Representative micrographs of HN137, HN159 and HN220 patient tumor derived primary and metastatic cultures treated with or without 0.25µM of bemcentinib (BGB324) or 0.25µM of barasertib for 72 hours. Cells were subsequently fixed and stained with 25% methanol and 0.5% crystal violet solution for visualization and quantification. Scale bar indicates 5000 µm. (Bottom) Quantification of surviving cells after drug treatment by determining the area stained with crystal violet using the ImageJ software. n=2 each for HN137 performed in 2 independent experiments, n=4 each for HN159 performed in 4 independent experiments and n=4 each for HN220 performed in 4 independent experiments. Red bar represents primary and blue bar represents metastatic. Data are presented as mean ± SEM. Each dot represents an individual sample. ***$p \leq 0.001$ indicate significant difference by unpaired two-tailed t test when compared to control, not corrected for multiple comparisons, ns not significant. Statistical analysis was not performed for **g** as the experiment was repeated only twice. **j** Gating strategy used to segregate cells of HN137, HN159 and HN220 expressing high (hi), middle (mid) or low or negative (lo/neg) of AXL or AURKB as determined by flow cytometry. **k** Primary (blue) or metastatic (ref) cells from HN137, HN159 and HN220 expressing high (hi), middle (mid) or low or negative (lo/neg) of AXL or AURKB as indicated on the x- and y-axes. n=3 pairs for HN137 performed in 3 independent experiments, n=15 primary and n=6 metastatic for HN159 performed in 5-6 independent experiments, and n=6 pairs for HN220 performed in 6 independent experiments. Black lines and error bars represent mean ± SEM. Red dot represents primary and blue dot represents metastatic. **l,m** Scatter plots showing the relationship between AXL or AURKB and EMT score of HNSCC primary tumors from patients, **l** regardless of nodal status (n=500), or **m** with no nodal metastasis only (n=171). **l,m** Data derived from TCGA. ρ = Pearson correlation with two-sided p-value; $p \leq 0.05$ indicates statistical significance as indicated within each scatterplot. **c,f-i,k** Source data are provided as a Source Data file.

Supplementary Figure 4

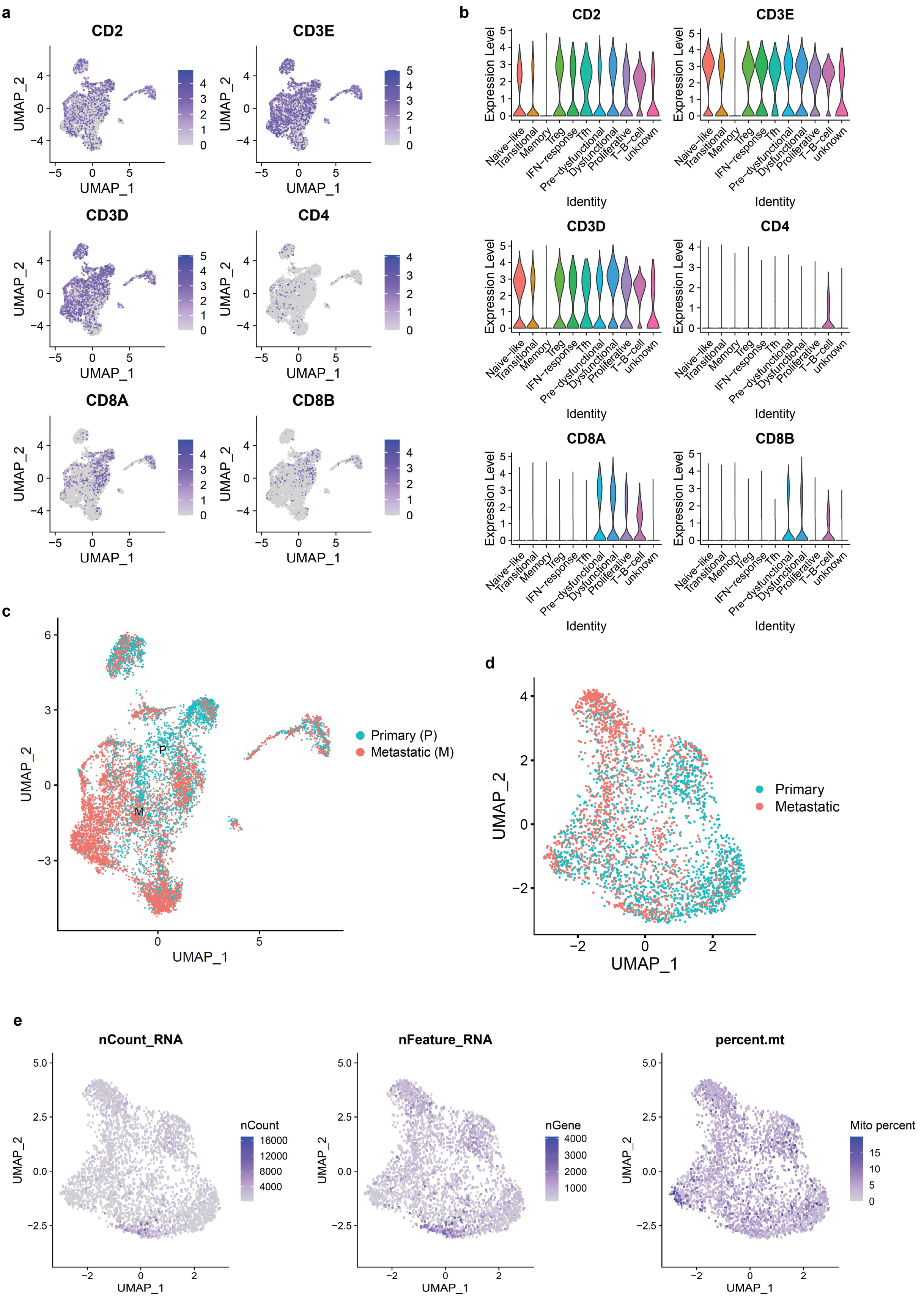

Supplementary Figure 4

Supplementary Figure 4. a 2D projection of selected genes for the annotation of T cell subpopulations in HNSCC tumors. N=10,168 cells. **b** Violin plots showing the expression level of selected genes for T cell annotation. **c** UMAP of tumor infiltrating T cells from HNSCC tumors. N=10,168 cells. **d** UMAP of CD8 T cells from HNSCC tumors. N=3,387 cells. **c,d** Clusters are denoted by tissue site of origin. **e** UMAP of CD8 T cells from **d**, denoted by (left) number of UMI counts, (mid) number of genes, and (right) mitochondrial gene percentage.

Supplementary Figure 5

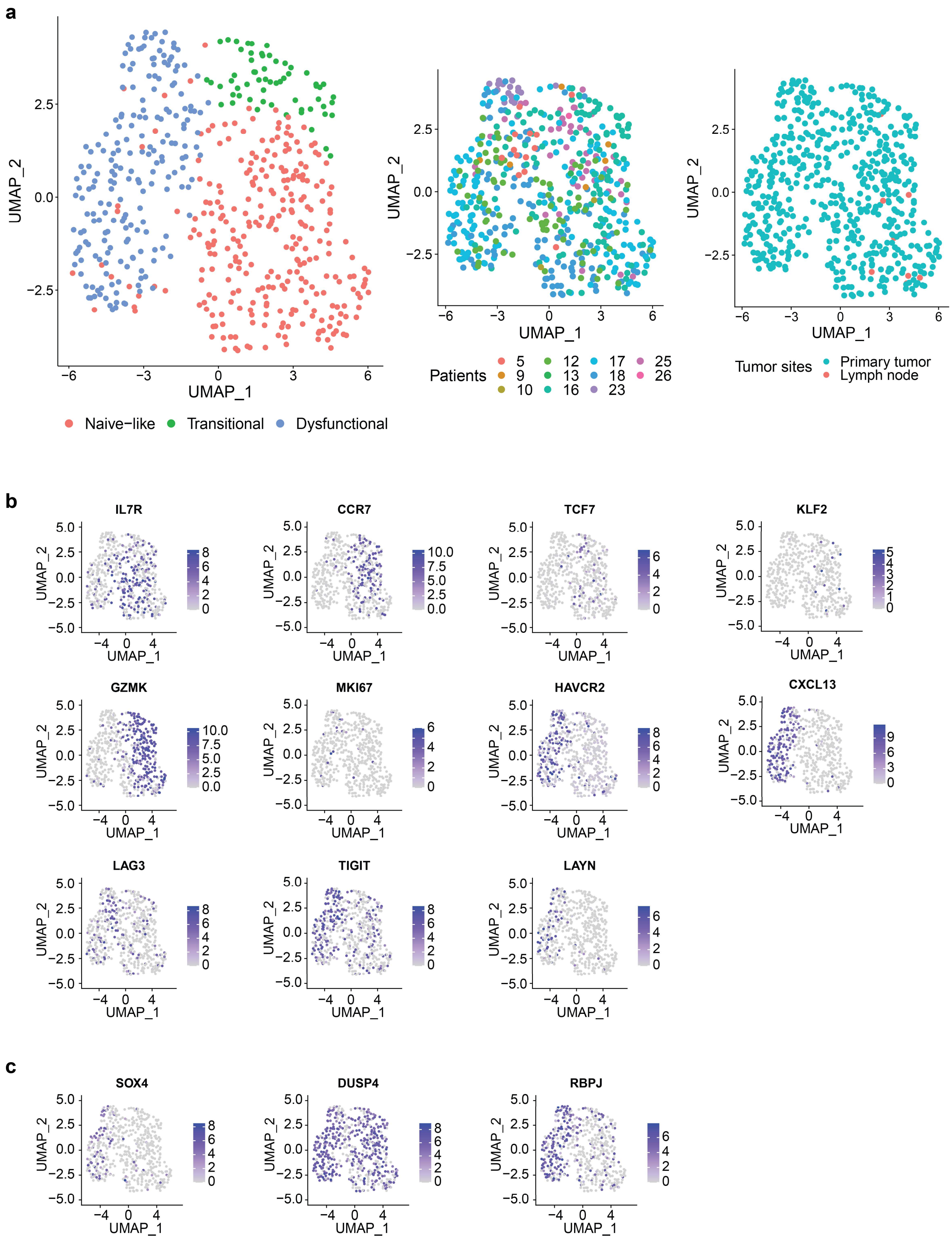

Supplementary Figure 5

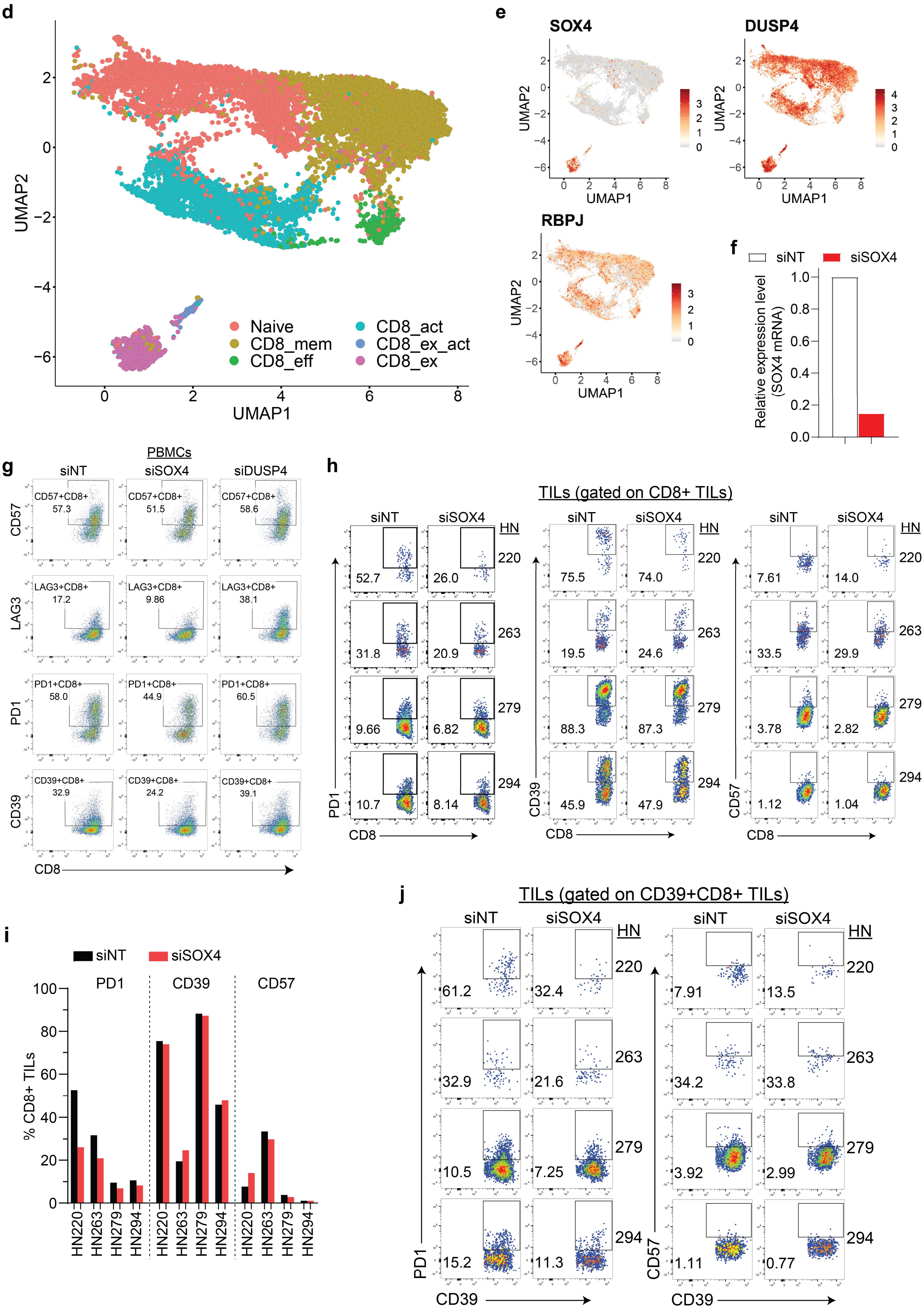

Supplementary Figure 5

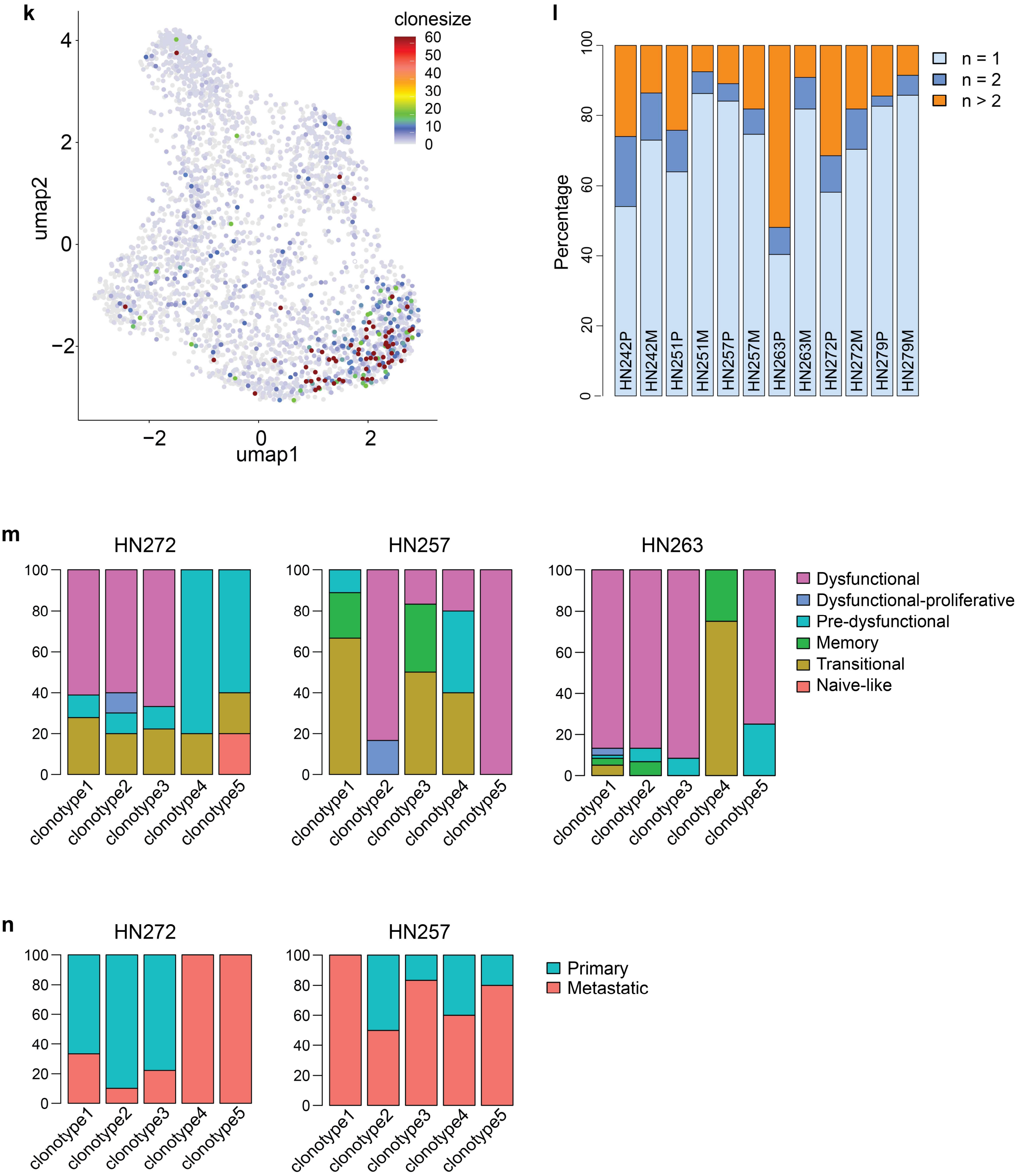

Supplementary Figure 5

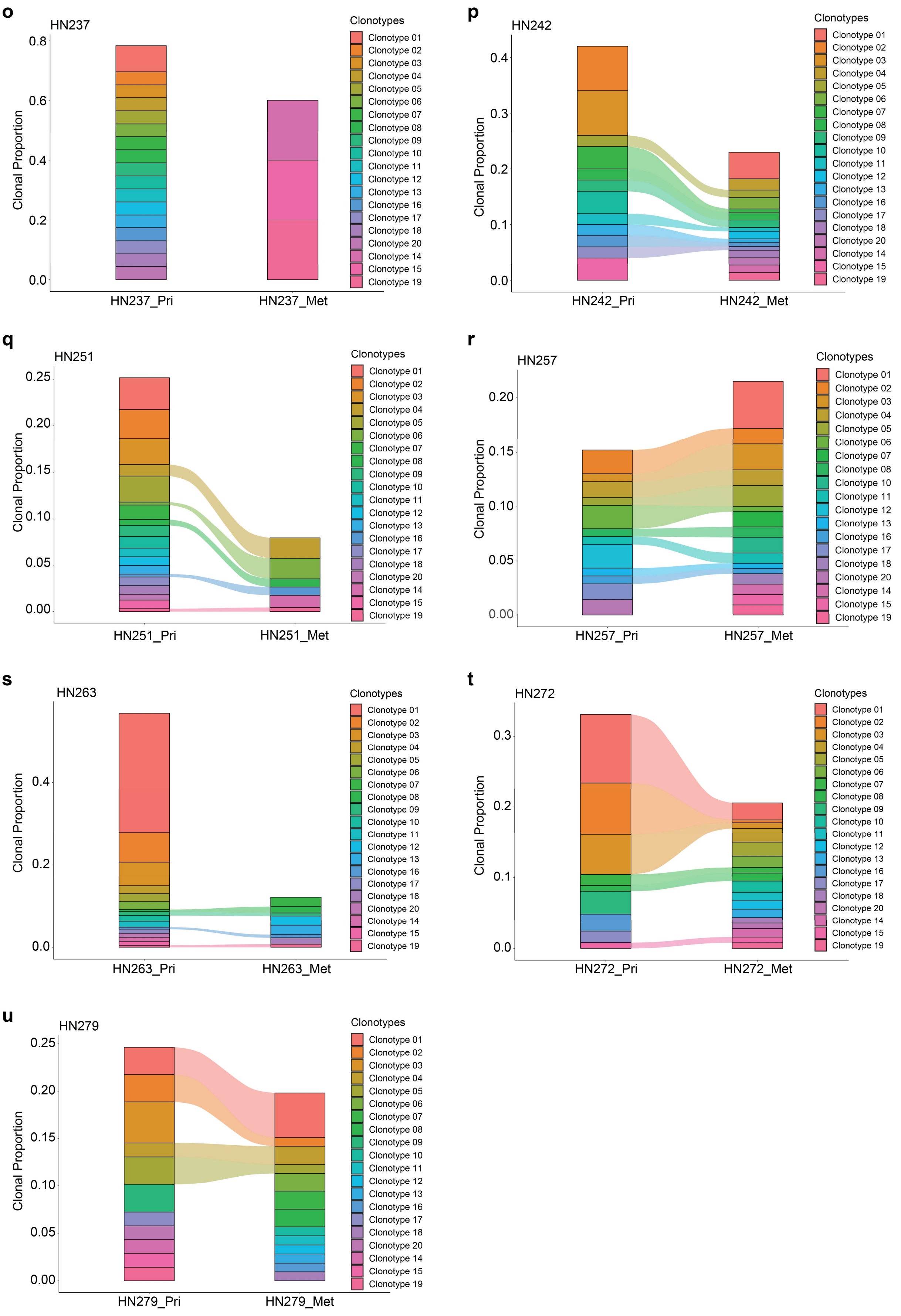

Supplementary Figure 5

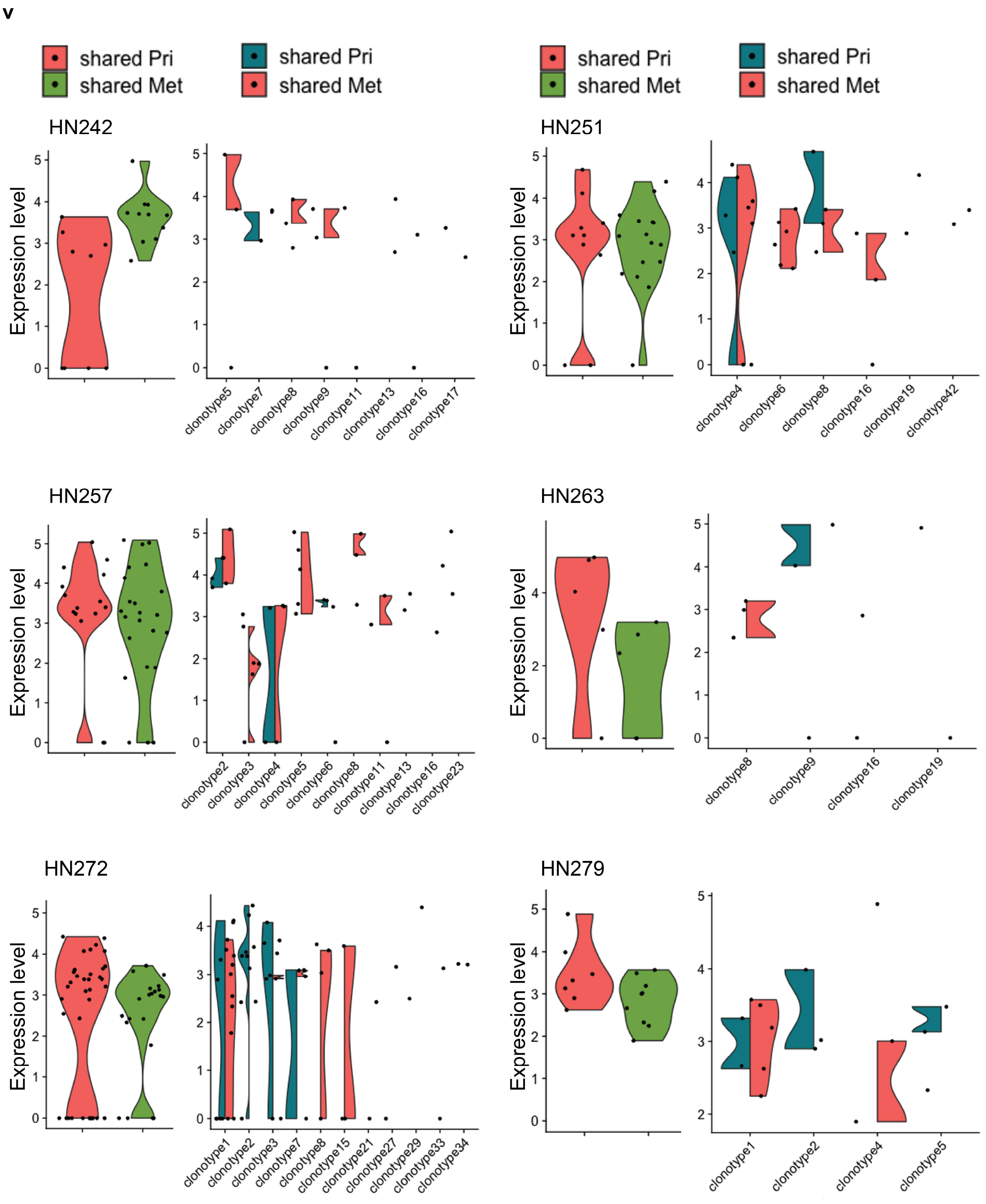

Supplementary Figure 5. a UMAP of tumor infiltrating CD8+ cells derived from Puram *et al*[1]. Cluster denotes (left) T cell state, (middle) sample ID and (right) site of origin of T cells. N=542 cells. **b** 2D projection of selected genes used to annotate T cell state in **a**. **c** UMAP projections of gene expression levels for SOX4, DUSP4 and RBPJ from **a**. **d** UMAP of tumor infiltrating T cells collected from cutaneous squamous cell carcinomas (SCC) samples treated with anti-PD1. scRNAseq meta-dataset derived from Yost KE and colleagues[2]. Clusters are denoted by colours and labelled with inferred cell identities. N=17,561 cells. **e** 2D projections of *SOX4*, *DUSP4* and *RBPJ* expression from **d**. **f** Relative mRNA expression of SOX4 in activated PBMC after 24 hour treatment with Accell siSOX4 or siNT quantified

by real-time PCR. **g** Representative flow cytometry plots showing the percentage of CD8 T cells expressing CD39, CD57, LAG3 or PD1 from PBMCs that were activated and cultured with siNT, siSOX4 or siDUSP4 for 5 days. **h** Flow cytometry plots and **i** bar plots showing the percentage of CD8 TILs expressing PD1, CD39 or CD57 that were activated and cultured with siNT or siSOX4 for 5 days. **j** Flow cytometry plots showing the percentage of CD39+CD8+ TILs expressing PD1 or CD57 from **h**. **k** UMAP of CD8 infiltrates from all patients colored by clone size. N=3,387 cells. **l** Percentage of TCR clone(s) detected once (n=1,399 cells), twice (n=182 cells) or more than two times (n=405 cells) across the samples. **m** TCR clonotype distribution across T cell differentiation states from tumors of HN272 (n=377), HN257 (n=347) and HN263 (n=340). **n** TCR clonotype distribution between primary or metastatic in HN272 (n=377) and HN257 (n=347). **m,n** Only the top 5 clonotypes from each sample are shown. **o-u** Alluvial diagrams showing the proportion of top 20 clonotypes between corresponding primary and metastatic tumors. Nodes (clonotypes) with links between tumor sites indicate clonotype sharing. **v** Maximum expression of gene *GZMB*, *GZMA*, *PRF1*, *IFNG*, *TNFA, CD69* or *TNFRSF9* in T cell clones with clonal sharing between primary and lymph nodes (metastatic) tumors of patients. Combined clonotypes (left) and individual clonotype (right) at each site for patient HN242 (n=20 cells), HN251 (n=24 cells), HN257 (n=36 cells), HN263 (n=10 cells), HN272 (n=48 cells), HN279 (n=16 cells). Each dot point represents one cell. **f,i** Source data are provided as a Source Data file.

Supplementary Figure 6

a

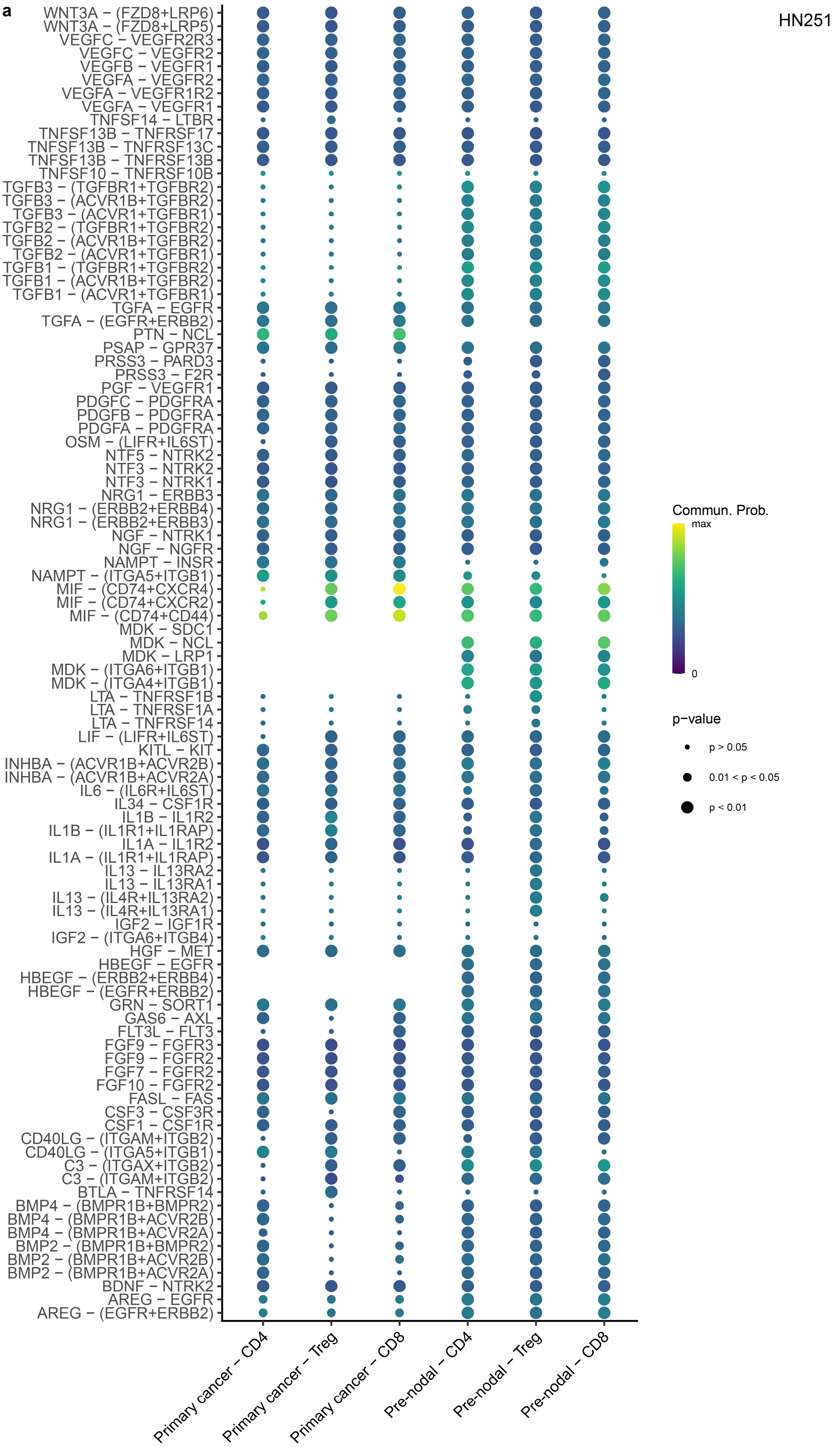

Supplementary Figure 6

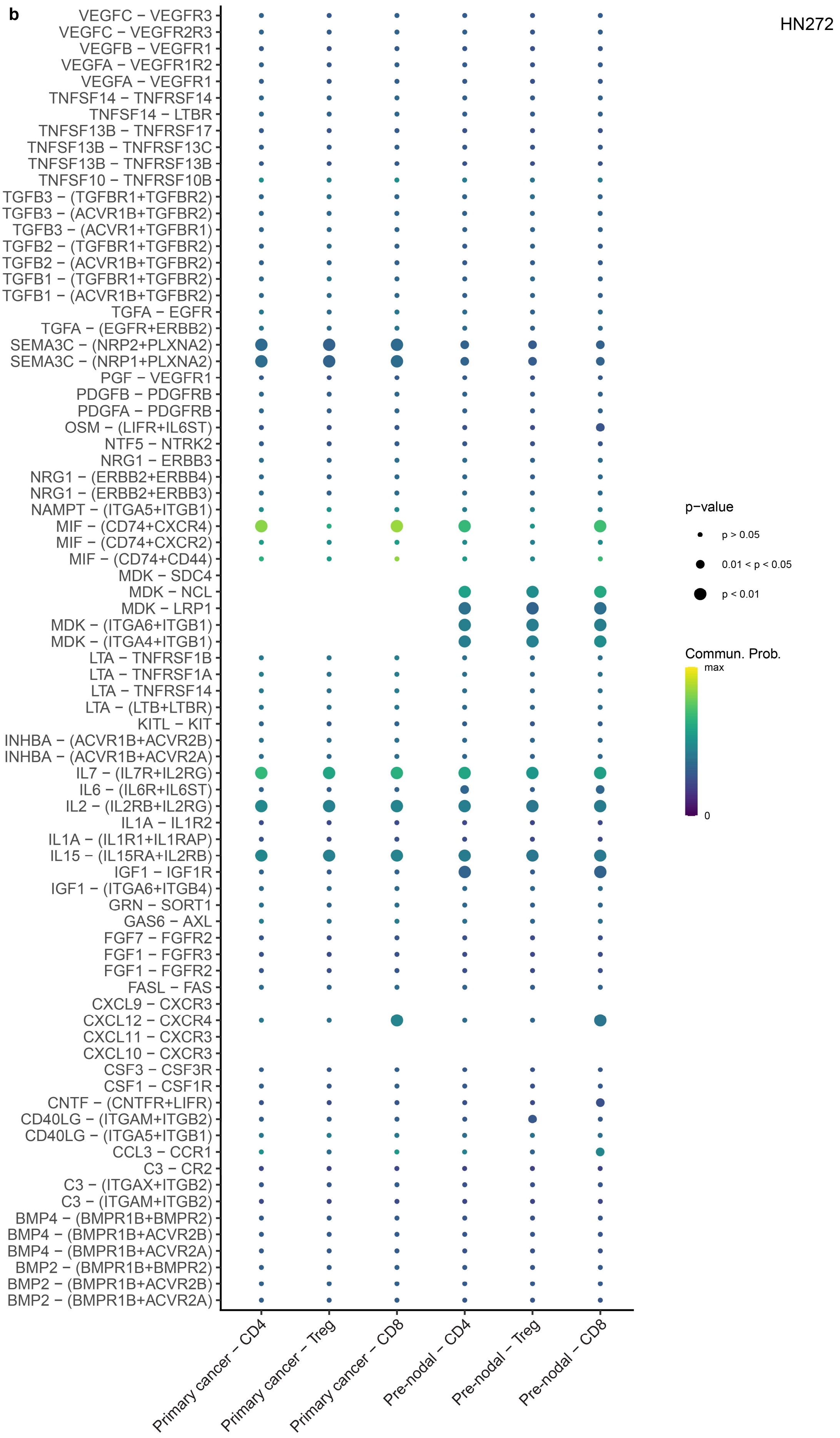

Supplementary Figure 6

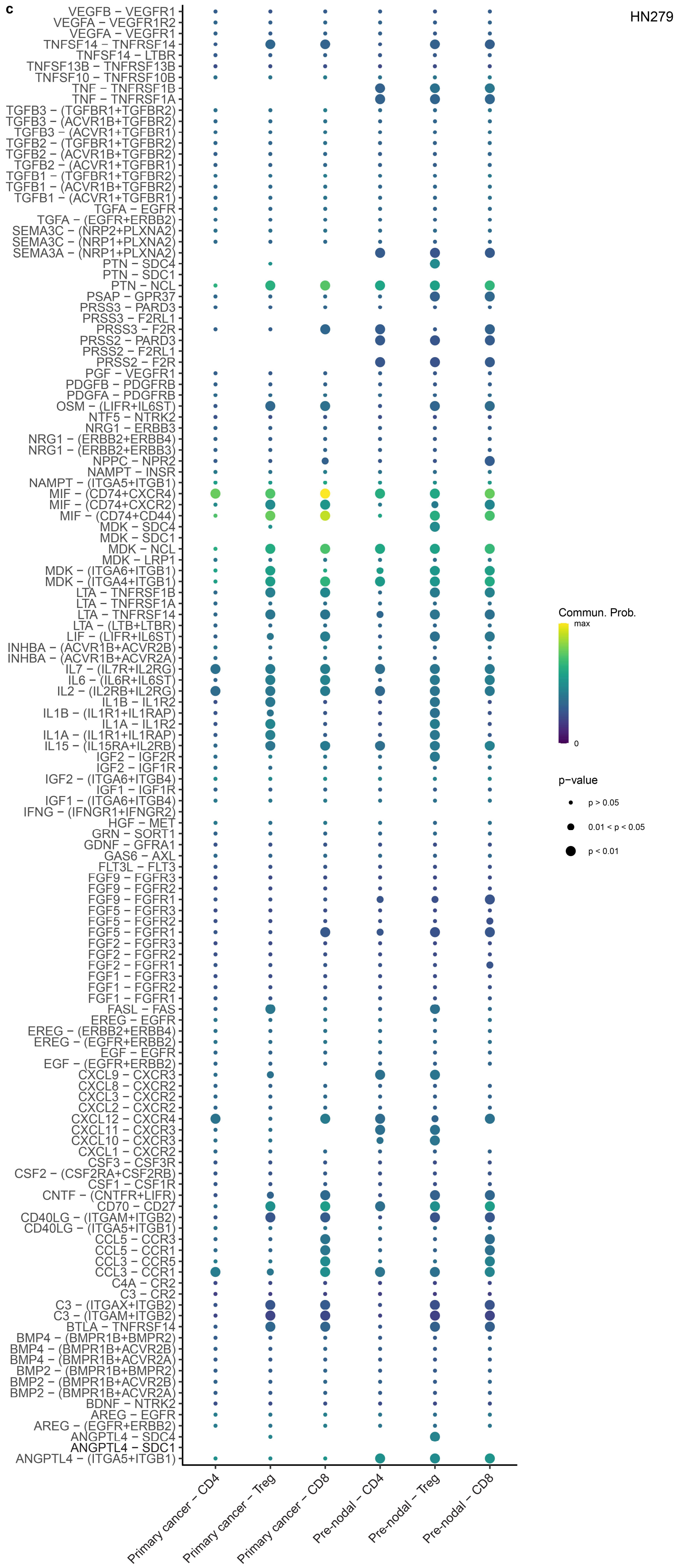

Supplementary Figure 6

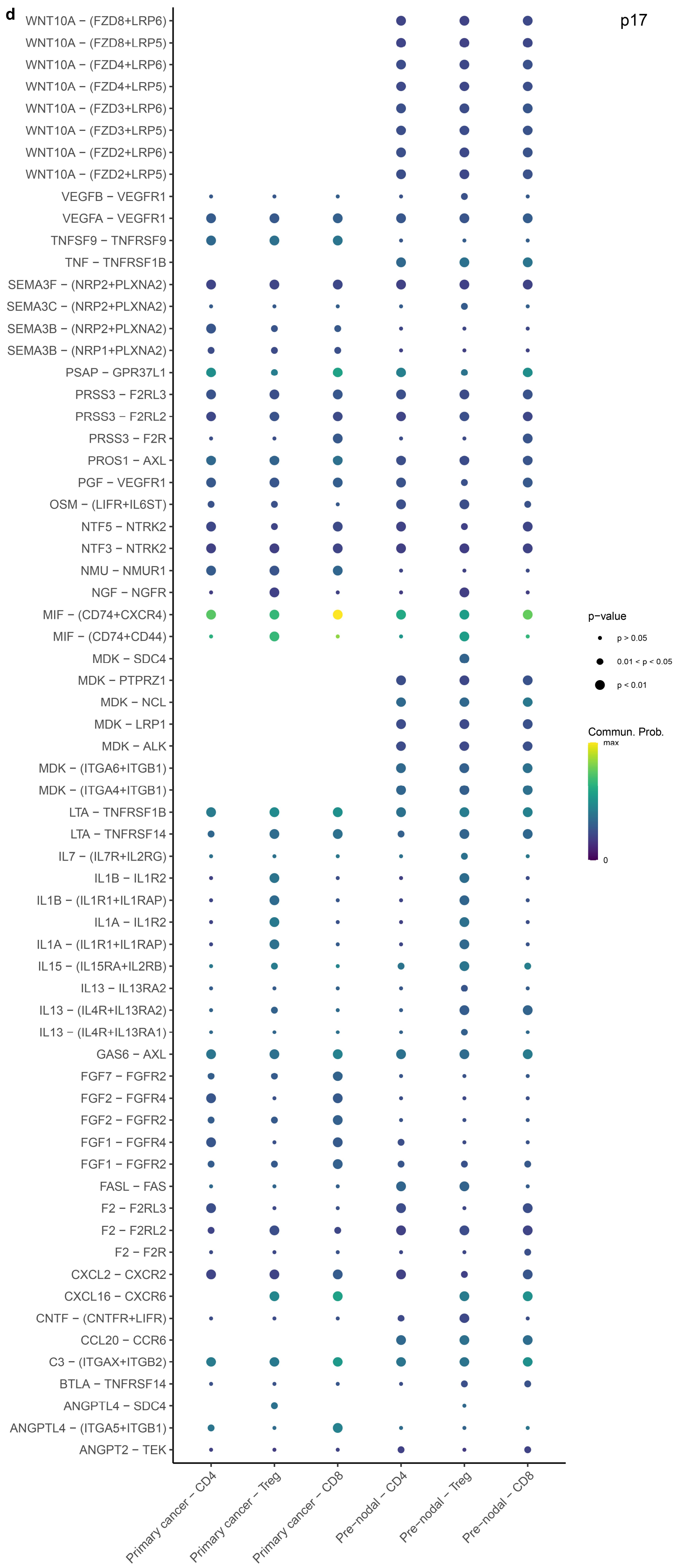

Supplementary Figure 6

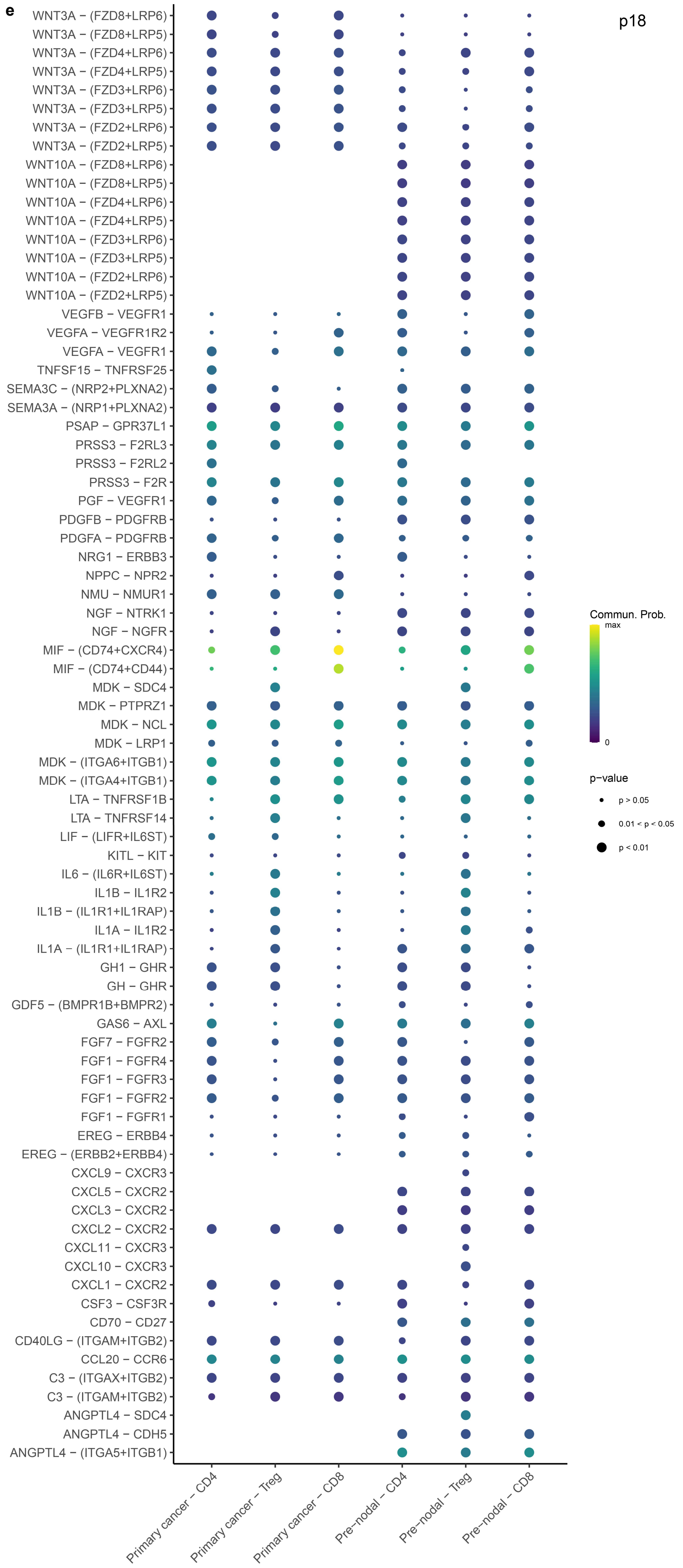

Supplementary Figure 6

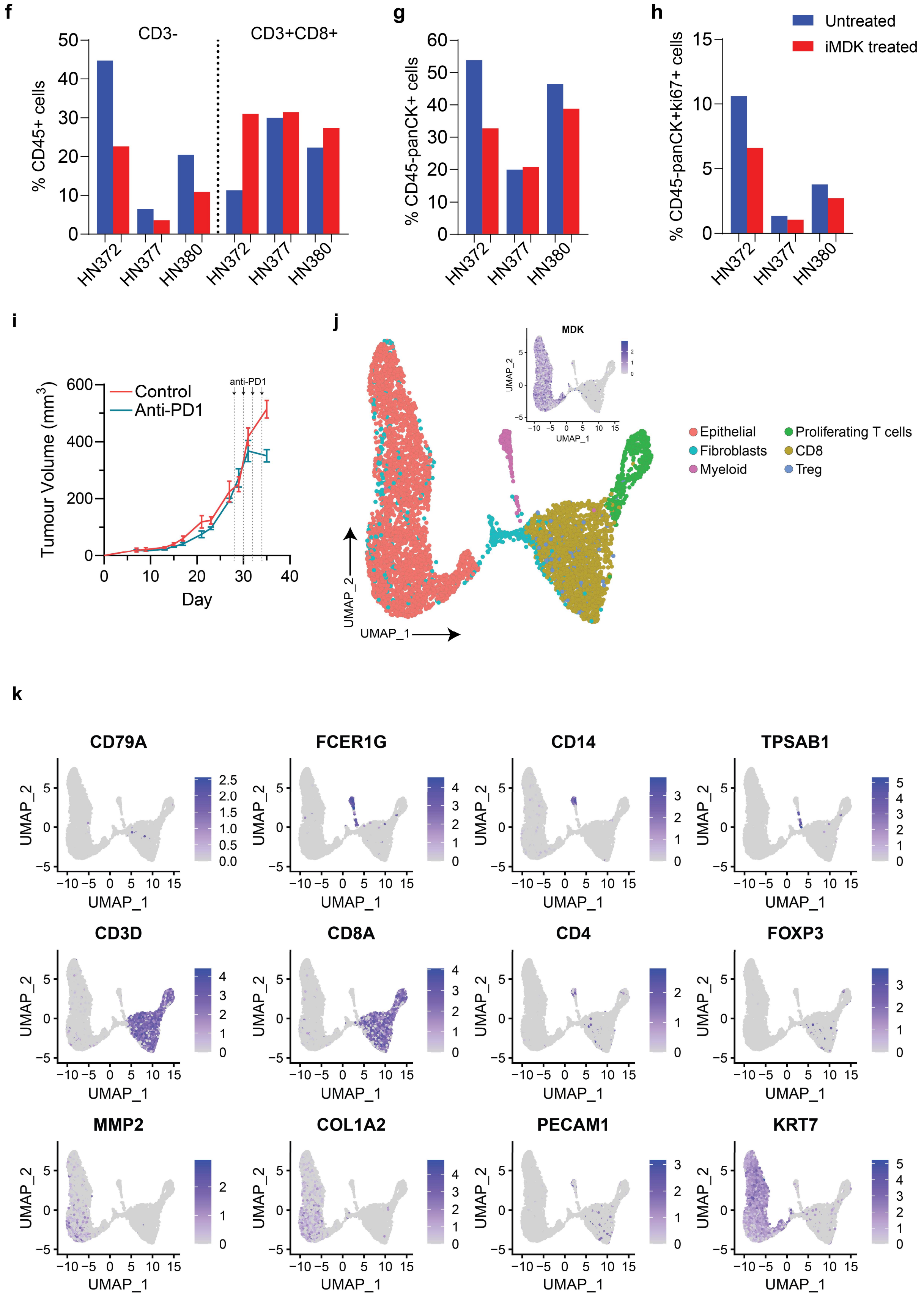

Supplementary Figure 6

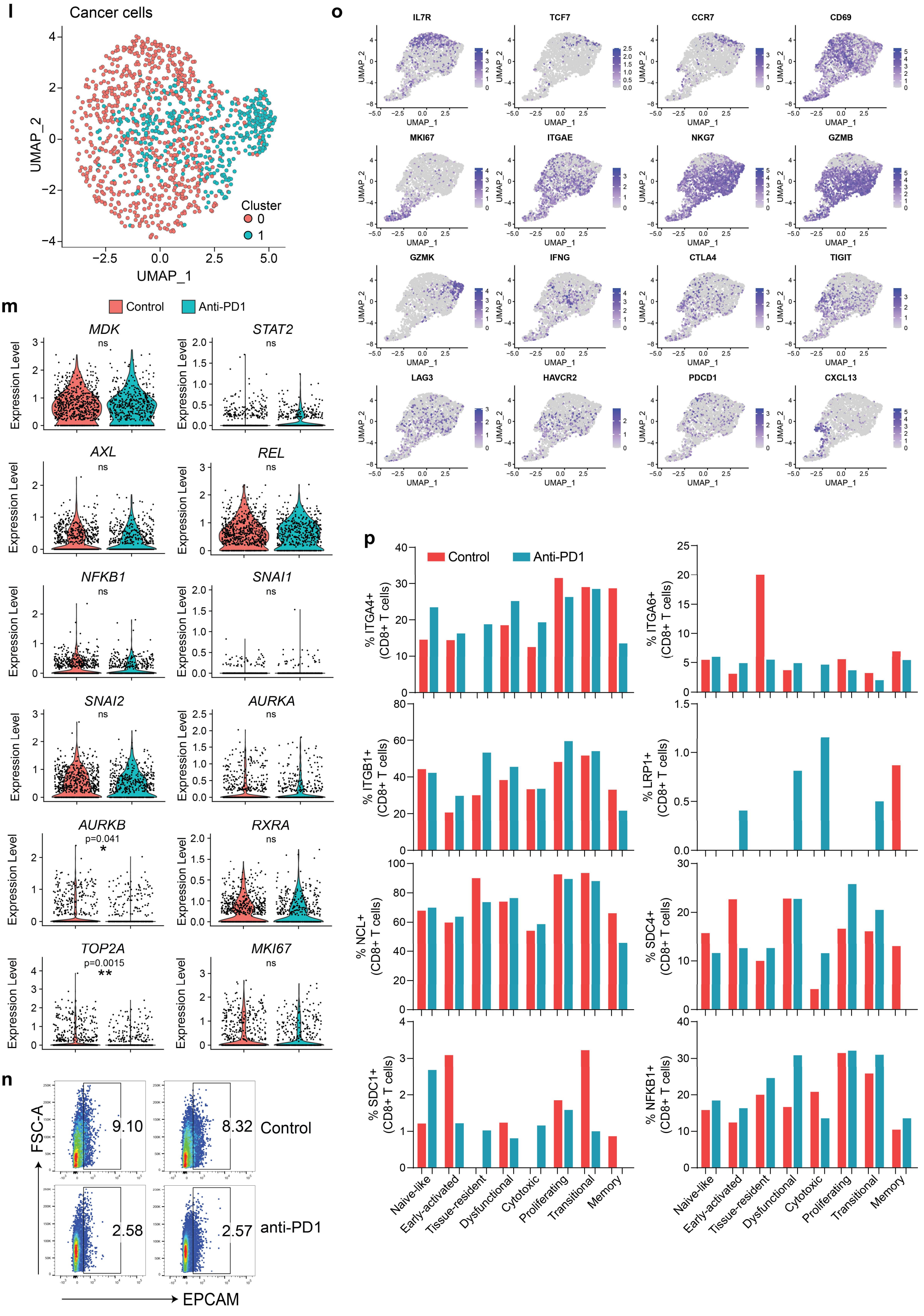

Supplementary Figure 6. a-e Cellchat dot plots showing significant ligand-receptor pairs contributing to the signalling from primary or pre-nodal cancer cells (epithelial) to Treg, CD4 or CD8 T cells in **a** HN251, **b** HN272, **c** HN279, **d** p17 and **e** p18. **d,e** Data for p17 and p18 were derived from Puram et al[1].The dot color and size represent the calculated communication probability. $p \leq 0.05$ indicates significant difference by one-sided permutation test as denoted on the right of each plot and the size of each dot. **f-h** Percentage of **f** CD3- and CD3+CD8+ T cells gated on CD45+ live cells, **g** CD45-panCK+ live cells and **h** CD45-panCK+ki67+ live cells in tumor cell suspensions after treatment with (red) or without (blue) MDK inhibitor for 5 days (n=3), analyzed by flow cytometry. **i** Mean tumor growth kinetic of humanized NOG-EXL mice treated with (blue) or without (red) anti-PD1 over time (n=4 each). Vertical grey dotted line and arrows indicate the days (28, 30, 32 and 34) when anti-PD1 was administered. Lines and error bars (blue and red) represent mean ± SEM. **j** UMAP of cells derived from tumors of humanized NOG-EXL mice treated with or without anti-PD1 (n=4 each; 6,261 cells). Clusters are denoted by colors labelled with inferred cell types, with a 2D projection of MDK gene expression (inset). **k** 2D projection of selected genes used annotation of cells captured from the humanized mouse experiment. **l** UMAP of all cancer cells derived from mice with or without anti-PD1 treatment (n=4 each; n=1,179 cells). **m** Violin plots showing the expression level of MDK and selected genes related to pre-metastatic progression in cancer cells and proliferation (identified in Supplementary Figure 2k). $^{*}p \leq 0.05$, $^{**}p \leq 0.01$ indicate significant difference by unpaired two-tailed t test when compared to control, ns not significant. **n** Representative dot plots showing frequency of EPCAM+ cells in tumors derived from control or anti-PD1 treated (bottom) humanized mice (n=2 each). Numerical figure within each dot plot indicates percentage. **o** 2D projection of selected genes for annotation of CD8 T cell subpopulations in tumors from humanized NOG-EXL mice. **p** Percentage of CD8 T cell subsets expressing the selected MDK receptor or NFKB1 genes. Source data are provided as a Source Data file.

Supplementary References

1. Puram, S. V. *et al.* Single-Cell Transcriptomic Analysis of Primary and Metastatic Tumor Ecosystems in Head and Neck Cancer. *Cell* **171**, 1611-1624 e1624, doi:10.1016/j.cell.2017.10.044 (2017).

2. Yost, K. E. *et al.* Clonal replacement of tumor-specific T cells following PD-1 blockade. *Nat Med* **25**, 1251-1259, doi:10.1038/s41591-019-0522-3 (2019).
