# Supplementary material for: Single cell analysis in head and neck cancer reveals potential immune evasion mechanisms during early metastasis
Source: Nat Commun. 2023 Mar 27;14:1680. doi: 10.1038/s41467-023-37379-y (PMC10042873; doi:10.1038/s41467-023-37379-y)
Supplement: Supplementary file 3 — Description of Additional Supplementary Files [file 41467_2023_37379_MOESM3_ESM.pdf]

Description of Additional Supplementary Files

File Name: Supplementary Data 1
Description: Samples included in this dataset.

File Name: Supplementary Data 2
Description: Clinical and pathologic feature of samples included in this dataset.

File Name: Supplementary Data 3
Description: List of 132 upregulated and 45 downregulated genes in HN257 subpopulations as described in Figure 2g. Log fold-change; p<0.05. p-values are calculated by two-sided Wilcoxon Rank Sum test and p-value adjustment is performed using bonferroni correction based on the total number of genes in the dataset.

File Name: Supplementary Data 4
Description: List of genes with log fold-change (p<0.05) of more than 2 or less than -2 when compared with HN137 pre-metastatic cancer cells vs HN137 primary cancer cells. q-values are calculated by two-sided Wilcoxon test followed by FDR correction to account for the number of genes tested.

File Name: Supplementary Data 5
Description: List of genes with log fold-change (p<0.05) of more than 2 or less than -2 when compared with HN159 pre-metastatic cancer cells vs HN159 primary cancer cells. q-values are calculated by two-sided Wilcoxon test followed by FDR correction to account for the number of genes tested.

File Name: Supplementary Data 6
Description: List of genes with log fold-change (p<0.05) of more than 2 or less than -2 when compared with HN220 pre-metastatic cancer cells vs HN220 primary cancer cells. q-values are calculated by two-sided Wilcoxon test followed by FDR correction to account for the number of genes tested.

File Name: Supplementary Data 7
Description: Cell ID, patient ID, tumor site, T cell type, cdr3 sequence and clonotypes for HN237. Only the top 20 clonotypes are highlighted (yellow).

File Name: Supplementary Data 8
Description: Cell ID, patient ID, tumor site, T cell type, cdr3 sequence and clonotypes for HN242. Only the top 20 clonotypes are highlighted (yellow).

File Name: Supplementary Data 9
Description: Cell ID, patient ID, tumor site, T cell type, cdr3 sequence and clonotypes for HN251. Only the top 20 clonotypes are highlighted (yellow).

File Name: Supplementary Data 10
Description: Cell ID, patient ID, tumor site, T cell type, cdr3 sequence and clonotypes for HN257. Only the top 20 clonotypes are highlighted (yellow).

File Name: Supplementary Data 11
Description: Cell ID, patient ID, tumor site, T cell type, cdr3 sequence and clonotypes for HN263. Only the top 20 clonotypes are highlighted (yellow).

File Name: Supplementary Data 12
Description: Cell ID, patient ID, tumor site, T cell type, cdr3 sequence and clonotypes for HN272. Only the top 20 clonotypes are highlighted (yellow).

File Name: Supplementary Data 13
Description: Cell ID, patient ID, tumor site, T cell type, cdr3 sequence and clonotypes for HN279. Only the top 20 clonotypes are highlighted (yellow).

File Name: Supplementary Data 14
Description: T cell receptor clustering with GLIPH2 analysis. TCR clonotype sharing across patients was observed in 5 of 126 clusters (highlighted in yellow or blue). The remaining clusters show sharing only within corresponding patient.
