# Supplementary material for: Single cell analysis in head and neck cancer reveals potential immune evasion mechanisms during early metastasis
Source: Nat Commun. 2023 Mar 27;14:1680. doi: 10.1038/s41467-023-37379-y (PMC10042873; doi:10.1038/s41467-023-37379-y)
Supplement: Supplementary file 18 — Reporting Summary [file 41467_2023_37379_MOESM18_ESM.pdf]

nature portfolio

Corresponding author(s): N Gopalakrishna Iyer

Last updated by author(s): Mar 13, 2023

Reporting Summary

Nature Portfolio wishes to improve the reproducibility of the work that we publish. This form provides structure for consistency and transparency in reporting. For further information on Nature Portfolio policies, see our Editorial Policies and the Editorial Policy Checklist.

Statistics

For all statistical analyses, confirm that the following items are present in the figure legend, table legend, main text, or Methods section.

n/a	Confirmed	
☐	☒	The exact sample size (*n*) for each experimental group/condition, given as a discrete number and unit of measurement
☐	☒	A statement on whether measurements were taken from distinct samples or whether the same sample was measured repeatedly
☐	☒	The statistical test(s) used AND whether they are one- or two-sided<br>*Only common tests should be described solely by name; describe more complex techniques in the Methods section.*
☒	☐	A description of all covariates tested
☐	☒	A description of any assumptions or corrections, such as tests of normality and adjustment for multiple comparisons
☐	☒	A full description of the statistical parameters including central tendency (e.g. means) or other basic estimates (e.g. regression coefficient) AND variation (e.g. standard deviation) or associated estimates of uncertainty (e.g. confidence intervals)
☐	☒	For null hypothesis testing, the test statistic (e.g. *F*, *t*, *r*) with confidence intervals, effect sizes, degrees of freedom and *P* value noted<br>*Give P values as exact values whenever suitable.*
☒	☐	For Bayesian analysis, information on the choice of priors and Markov chain Monte Carlo settings
☒	☐	For hierarchical and complex designs, identification of the appropriate level for tests and full reporting of outcomes
☐	☒	Estimates of effect sizes (e.g. Cohen's *d*, Pearson's *r*), indicating how they were calculated

Our web collection on statistics for biologists contains articles on many of the points above.

Software and code

Policy information about availability of computer code

Data collection	All code used to analyse the dataset is openly available at https://doi.org/10.5281/zenodo.7692887. All software and algorithms used in this study are publicly available and are listed in the Methods section.
Data analysis	10x droplet-based single cell RNA-seq data were performed using Seurat version 3.1.5. InferCNV v1.2.2 (https://github.com/broadinstitute/inferCNV) was used to identify somatic large-scale chromosomal copy number alterations using single cell gene expression data. CopyKAT v1.0.8 (https://github.com/navinlabcode/copykat) was used to infer copy number profiles and assign diploid/aneuploid labels to each cell. Monocle v2.16.0 R package were used to detect branches and states. CytoTRACE v0.3.2 R package (https://cytotrace.stanford.edu/) was applied to predict the differentiation score and plotted on the monocle trajectories. GeneSwitches v0.1.0 R package (https://github.com/SGDDNB/GeneSwitches) was applied to determine the significant genes that regulate the trajectory from primary to pre-metastatic cells. scde v1.99.2 R package (https://github.com/hms-dbmi/scde) were used to apply pathway and gene set overdispersion analysis (PAGODA) for gene ontologies analysis and clustering. Slingshot v1.4.0 R package (https://github.com/kstreet13/slingshot) was used to performed trajectory analyses of the CD8+ T-cells.<br><br>For C1 microfluidic-based single-cell RNA-seq, quantification of gene expression was determined using RSEM v1.3.0 (https://github.com/deweylab/RSEM) to generate TPM values.<br><br>The RNA-seq profiles from the TCGA data were deconvolved using xCell (https://portal.gdc.cancer.gov/).<br><br>FlowJo v10.5.3, Graphpad Prism 8.0.2, inForm software (version 2.4.2), ImageJ 1.53c.

Further details can be found in the Methods of the manuscript.

For manuscripts utilizing custom algorithms or software that are central to the research but not yet described in published literature, software must be made available to editors and reviewers. We strongly encourage code deposition in a community repository (e.g. GitHub). See the Nature Portfolio guidelines for submitting code & software for further information.

Data

Policy information about availability of data

All manuscripts must include a data availability statement. This statement should provide the following information, where applicable:

- Accession codes, unique identifiers, or web links for publicly available datasets
- A description of any restrictions on data availability
- For clinical datasets or third party data, please ensure that the statement adheres to our policy

For 10x droplet-based single cell RNAseq, reads were aligned to the GRCh38 reference genome and quantified using cellranger count version 2.2.0 (10x Genomics, Pleasanton, CA). TCR reads were mapped to vdj_GRCh38_alts_ensembl-3.1.0-3.1.0 reference genome and quantified using cellranger count version 3.1.0 (10x Genomics, Pleasanton, CA). For C1 microfluidic-based single-cell RNAseq, the raw reads in FASTQ files were aligned to the human genome (hg19 assembly) using STAR v2.6.0.

The raw human and mouse 10x scRNAseq raw data generated in this study and the corresponding processed Seurat objects with all cells have been deposited in the GEO under accession code GSE188737 [https://www.ncbi.nlm.nih.gov/geo/query/acc.cgi?acc=GSE188737] and GSE225170 [https://www.ncbi.nlm.nih.gov/geo/query/acc.cgi?acc=GSE225170] respectively. The Fluidigm C1 scRNA-seq raw data and processed gene expression matrix have been deposited in the GEO under accession code GSE225331 [https://www.ncbi.nlm.nih.gov/geo/query/acc.cgi?acc=GSE225331]. The scRNAseq count data and original cell annotations for the published study of HNSCC (Puram SV et al, 2017, Cell) and cutaneous squamous-cell carcinoma (Yost KE et al, 2019, Nature Medicine) were downloaded from the GEO under accession code GSE103322 [https://www.ncbi.nlm.nih.gov/geo/query/acc.cgi?acc=GSE103322] and GSE123813 [https://www.ncbi.nlm.nih.gov/geo/query/acc.cgi?acc=GSE123813]. The HNSCC transcriptome profiling gene expression files with FPKM values and related clinical parameters were obtained from TCGA portal (https://cancergenome.nih.gov). The remaining data are available within the Article, Supplementary Information or Source Data file.

10x scRNAseq data for tumor cells and CD8+ T-cells can be accessed and interrogated as an interactive web application via the following Shiny app (http://hnc.ddnetbio.com/).

TCGA RNAseq and clinical annotation data were downloaded from the GDC Data Portal [https://portal.gdc.cancer.gov/].

The remaining data are available within the Article, Supplementary Information or Source Data file.

Human research participants

Policy information about studies involving human research participants and Sex and Gender in Research.

Reporting on sex and gender	We did not perform or report analysis based on sex and gender in our study as this information was not relevant to the study. Both females and males were used in our dataset without any prejudice.
Population characteristics	For tumor samples, covariant-relevant population characteristics including clinical and pathologic features are provided in Supplementary data 1 and 2; subjects included both males and females, aged 21-85.<br><br>Umbilical cord blood samples were collected from uncomplicated pregnancies at term and patients were recruited from planned Cesarean deliveries without any prejudice.
Recruitment	For tumor samples, all patients were confirmed histologically to be HNSCC and suitable for surgical resection (with no prior cancer treatment).<br><br>Umbilical cord blood samples were collected from uncomplicated pregnancies at term and patients were recruited from planned Cesarean deliveries without any prejudice.
Ethics oversight	The study of patient tumor samples was approved by SingHealth Centralized Institutional Review Board (CIRB: 2014/2093, 2018/2512 and 2016/2757) and each patient’s parent(s) written consent.<br><br>Human cord blood samples were collected at the KK Women’s and Children’s Hospital, Singapore, and were performed in compliance with Institutional Review Board (CIRB Ref: 2013/778/D and 2019/2443/D) and each patient’s parent(s) written consent for donation of cord blood for the generation of humanized mouse models.

Note that full information on the approval of the study protocol must also be provided in the manuscript.

Field-specific reporting

Please select the one below that is the best fit for your research. If you are not sure, read the appropriate sections before making your selection.

[x] Life sciences [ ] Behavioural & social sciences [ ] Ecological, evolutionary & environmental sciences

Life sciences study design

All studies must disclose on these points even when the disclosure is negative.

Sample size	No statistical method was used to determine the patient sample size. The number of patient samples included in this study was primarily determined by the availability of samples obtained. However, the sample size in this study are consistent with prior single-cell studies. For 10X single-cell cell number, we attempted to obtain greater than 500 cells per patient or per mouse treatment cohort. For C1 single-cell cell number, we attempted to obtain greater than 80 cells per sample.
Data exclusions	No data were excluded from analysis.
Replication	Single cell and bulk sequencing data from patients were not suitable for replication. For functional experiments, at least n=2 of data point was performed from at least 2 independent experiments and all attempts at replication were successful.
Randomization	No randomization was used for patient samples since no treatment was given. Randomization was used for humanized mouse samples since 50% were used for anti-PD1 treatment and 50% were used as controls.
Blinding	No blinding was used for patient identity since no treatment was given. For experiments other than those involving patients, no blinding was used due the requirement of prior knowledge of samples (ie. primary or metastatic lymph nodes cell culture; viral and nodal status of external samples) and treatment status to perform analyses.

Reporting for specific materials, systems and methods

We require information from authors about some types of materials, experimental systems and methods used in many studies. Here, indicate whether each material, system or method listed is relevant to your study. If you are not sure if a list item applies to your research, read the appropriate section before selecting a response.

Materials & experimental systems

n/a	Involved in the study	
☐	☒	Antibodies
☐	☒	Eukaryotic cell lines
☒	☐	Palaeontology and archaeology
☐	☒	Animals and other organisms
☒	☐	Clinical data
☒	☐	Dual use research of concern

Methods

n/a	Involved in the study	
☒	☐	ChIP-seq
☐	☒	Flow cytometry
☒	☐	MRI-based neuroimaging

Antibodies

Antibodies used

Antibodies used included antibody recognizing
AURKB (clone RM278, cat. #MA5-27890, lot #UL2892951, Invitrogen); 1:200 dilution),
AXL (clone C89E7 cat. #8661S, lot #6, Cell Signaling Technology; 1:200 dilution),
AXL conjugated to APC (clone #108724, cat. #FAB154A, lot #ADDW0217051, R&D Systems; 1:200 dilution),
CD57 conjugated to FITC (clone HNK-1, cat. #322306, lot #B241665; 1:50 dilution),
LAG3 conjugated to PE-Cy7 (clone 11C3C65, cat. #369310, lot #B289010, Biolegend; 1:50 dilution),
CD39 conjugated to BV421 (clone A1, cat. #328214, lot #B242450, Biolegend; 1:50 dilution),
CD4 conjugated to PE (clone OKT4, cat. #317410, lot #B211218, Biolegend; 1:50 dilution),
PD1 conjugated to APC (clone J105, cat. #17-2799-42, lot #2010675, eBioscience; 1:50 dilution),
CD8 conjugated to APC-eFluor 780 (clone SK1, cat. #47-0087-42, lot #2255548, eBioscience; 1:50 dilution),
CD4 conjugated to PE-Cy7 (clone SK3, cat. 557852, lot #0111321, BD Biosciences; 1:50 dilution),
human CD45 conjugated to PerCpCy5.5 (clone HI30, cat. #45-0459-42, eBioscience; 1:50 dilution),
mouse CD45 conjugated to eFlour450 (clone 30-F11, cat. #48-0451-82, eBioscience; 1:50 dilution),
EpCAM conjugated to PE (clone HEA-125, cat. #130-113-826, Miltenyi Biotech; 1:50 dilution),
CD3 conjugated to FITC (clone OKT3, cat. #317306, lot #B327673, Biolegend; 1:50 dilution),
Ki67 conjugated to PE (clone B56, cat. #556027, lot #356079, BD Biosciences; 1:5 dilution),
pan-cytokeratin conjugated to AF488 (clone AE1/AE3, cat. #53-9003-82, lot #2290741, eBioscience; 1:100 dilution),
human CD45 conjugated to PerCpCy5.5 (clone HI30, cat. #304028, lot #B257291, Biolegend; 1:50 dilution),
goat anti-rabbit IgG secondary antibody conjugated to AF647 (cat. #A32733; lot #UF277970, Invitrogen; 1:200 dilution),
goat anti-rabbit IgG secondary antibody conjugated to AF488 (cat. #A11008; lot #1408830, Invitrogen; 1:200 dilution),
mouse IgG1 isotype antibody conjugated to APC (clone MOPC-21, cat. #555751, BD Biosciences; 1:200 dilution) and
rabbit IgG1 isotype antibody (clone DA1E, cat. #3900S, lot #37, R&D systems; 1:500 dilution).

Validation

All antibodies were validated by the manufacturer, and antibody-specific staining was compared to isotype control when necessary.

The following antibodies were validated by “Advanced verification” by the manufacturer to ensure that the antibody binds to the antigen stated:
Rabbit anti-human AURKB (clone RM278, cat. #MA5-27890, lot #UL2892951, Invitrogen),
Mouse anti-human Pan-cytokeratin conjugated to AF488 (clone AE1/AE3, cat. #53-9003-82, lot #2290741, eBioscience).

The following mouse anti-human antibodies were validated by isotype control antibodies by flow cytometry:
AXL conjugated to APC (clone #108724, cat. #FAB154A, lot #ADDW0217051, R&D Systems),
CD57 conjugated to FITC (clone HNK-1, cat. #322306, lot #B241665, Biolegend),
LAG3 conjugated to PE-Cy7 (clone 11C3C65, cat. #3969310, lot #B289010, Biolegend),
CD39 conjugated to BV421 (clone A1, cat. #328214, lot #B242450, Biolegend),
CD4 conjugated to PE (clone OKT4, cat. #317410, lot #B211218, Biolegend),
CD8 conjugated to APC-eFluor 780 (clone SK1, cat. #47-0087-42, lot #2255548, eBioscience),
CD4 conjugated to PE-Cy7 (clone SK3, cat. 557852, lot #0111321, BD Biosciences),
human CD45 conjugated to PerCpCy5.5 (clone HI30, cat. #45-0459-42, eBioscience),
CD3 conjugated to FITC (clone OKT3, cat. #317306, lot #B327673, Biolegend),
Ki67 conjugated to PE (clone B56, cat. #556027, lot #356079, BD Biosciences),
human CD45 conjugated to PerCpCy5.5 (clone HI30, cat. #304028, lot #B257291, Biolegend).

Mouse anti-human EpCAM conjugated to PE (clone HEA-125, cat. #130-113-826, Miltenyi Biotech) was validated by staining with normal human peripheral blood leukocytes.

Rabbit anti-human AXL (clone C89E7 cat. #8661S, lot #6, Cell Signaling Technology) was validated by staining multiple paraffin-embedded cancer tissues by immunohistochemistry.

Mouse anti-human PD1 conjugated to APC (clone J105, cat. #17-2799-42, lot #2010675, eBioscience) was validated by staining 3-day PHA-stimulated human peripheral blood cells by flow cytometry.

Rat anti-mouse CD45 conjugated to eFlour450 (clone 30-F11, cat. #48-0451-82, eBioscience) was validated by isotype control antibodies by flow cytometry.

Eukaryotic cell lines

Policy information about cell lines and Sex and Gender in Research

Cell line source(s)	All cell lines were patient-derived tumor cell cultures that were passaged until a majority of tumor cells was observed.
Authentication	Cell line identity was authenticated by comparing the STR profile (Indexx BioResearch), mutational and/or expression profile of each cell line to its original tumour.
Mycoplasma contamination	All lines were tested and confirmed to be free of mycoplasma at the time of experiments.
Commonly misidentified lines (See ICLAC register)	All cell lines were derived directly from patient tumors and passaged until a majority of tumor cells was observed. No genetic manipulation was performed.

Animals and other research organisms

Policy information about studies involving animals; ARRIVE guidelines recommended for reporting animal research, and Sex and Gender in Research

Laboratory animals	Six to eight weeks old NOG-EXL (hGM-CSF/hIL-3 NOG) female mice (n=16) were used for human CD34+ hematopoietic stem cells pre-engraftment. Sixteen weeks later, these mice were treated with or without a course of Pembrolizumab. Mice were housed in a 12 light/12 dark cycle at approximately 18-23 degree Celsius with 40-60% humidity.
Wild animals	The study did not involve wild animals.
Reporting on sex	We did not perform or report analysis based on sex and gender as this information was not relevant to the study. Only female mice were available and used for the experiment.
Field-collected samples	The study did not involve samples collected from the field.
Ethics oversight	All animal experiments were approved by the Institutional Animal Care and Use Committee of the Biological resource centre (BRC), A*STAR, Singapore (IACUC numbers 161192, 191496)

Note that full information on the approval of the study protocol must also be provided in the manuscript.

Flow Cytometry

Plots

Confirm that:

☒ The axis labels state the marker and fluorochrome used (e.g. CD4-FITC).

☒ The axis scales are clearly visible. Include numbers along axes only for bottom left plot of group (a 'group' is an analysis of identical markers).

☒ All plots are contour plots with outliers or pseudocolor plots.

☒ A numerical value for number of cells or percentage (with statistics) is provided.

Methodology

Sample preparation	For cell surface staining, patient-derived cell lines were trypsinized into single cell suspensions and cultured PBMCs were stained with Fixable Viability Dye eFluor 506 (cat. #65-0866-14, eBioscience) and fluorochrome conjugated antibodies for 30-60 mins on ice in the dark. Washes were performed using ice-cold 1% BSA in PBS.<br><br>For intracellular staining, trypsinized cells were first stained with Fixable Viability Dye eFluor 506 (cat. #65-0866-14, eBioscience) and antibodies recognizing surface antigens, and subsequently fixed and permeabilized with an eBioscience Foxp3/Transcription Factor Staining Buffer Set according to the manufacturer protocol. All staining steps were performed for 30-60 mins on ice in the dark.
Instrument	All samples were acquired using a BD FACSCanto II or BD LSRFortressa, and sorted using a BD FACSAria III.
Software	Flow cytometry data was analyzed using Flowjo.
Cell population abundance	Post-sort purities were assessed by flow cytometry and confirmed to be > 95%. Post-sort purities were also validated by invasion assays
Gating strategy	For the cell line experiment, cells were gated on FSC-A/SSC-A, and then gated to exclude doublets based on FSC-A/FSC-H. Dead cells were excluded based on Fixable Viability Dye eFluor 506 (cat. #65-0866-14, eBioscience). Cellular expression of AXL or AURKB were categorized as high, middle or low/negative. An example of the gating strategy used is shown in Supplementary Figure 3J.<br><br>For the gene knock-down experiment, cells were gated on FSC-A/SSC-A, and then gated to exclude doublets based on FSC-A/FSC-H. Dead cells were excluded based on Fixable Viability Dye eFluor 506 (cat. #65-0866-14, eBioscience). Cells were then gated for CD8+ cells (CD8+CD4-) and then categorized as CD8 cells positive for CD57, LAG3, PD1 or CD39. An example of the gating strategy used is shown in Supplementary Figure 5g-h & j.<br><br>For the Midkine inhibition experiment, cells were gated on FSC-A/SSC-A, and then gated to exclude doublets based on FSC-A/FSC-H. Dead cells were excluded based on Fixable Viability Dye eFluor 506 (cat. #65-0866-14, eBioscience). Cells were then gated for CD45+ immune cells (CD3+CD8+, CD3+CD8-, CD3-CD8-) or CD45- cells (panCK+, panCK+ki67+). An example of the gating strategy used is shown in Figure 6c.

☒ Tick this box to confirm that a figure exemplifying the gating strategy is provided in the Supplementary Information.
